# Biochars from Olive Stones as Carbonaceous Support in Pt/TiO_2_-Carbon Photocatalysts and Application in Hydrogen Production from Aqueous Glycerol Photoreforming

**DOI:** 10.3390/nano13091511

**Published:** 2023-04-28

**Authors:** Juan Carlos Escamilla-Mejía, Jesús Hidalgo-Carrillo, Juan Martín-Gómez, Francisco J. López-Tenllado, Rafael Estévez, Alberto Marinas, Francisco J. Urbano

**Affiliations:** Departamento de Química Orgánica, Instituto Químico para la Energía y el Medioambiente (IQUEMA), Edificio Marie Curie, Campus de Rabanales, Universidad de Córdoba, E-14071 Córdoba, Spain; qo2esmej@uco.es (J.C.E.-M.); q92magoj@uco.es (J.M.-G.); b42lotef@uco.es (F.J.L.-T.); q72estor@uco.es (R.E.); alberto.marinas@uco.es (A.M.)

**Keywords:** glycerol photoreforming, hydrogen production, Pt/TiO_2_-carbon, photodeposition, deactivation

## Abstract

Several biochars were synthesized from olive stones and used as supports for TiO_2_, as an active semiconductor, and Pt as a co-catalyst (Pt/TiO_2_-PyCF and Pt/TiO_2_-AC). A third carbon-supported photocatalyst was prepared from commercial mesoporous carbon (Pt/TiO_2_-MCF). Moreover, a Pt/TiO_2_ solid based on Evonik P25 was used as a reference. The biochars used as supports transferred, to a large extent, their physical and chemical properties to the final photocatalysts. The synthesized catalysts were tested for hydrogen production from aqueous glycerol photoreforming. The results indicated that a mesoporous nature and small particle size of the photocatalyst lead to better H_2_ production. The analysis of the operational reaction conditions revealed that the H_2_ evolution rate was not proportional to the mass of the photocatalyst used, since, at high photocatalyst loading, the hydrogen production decreased because of the light scattering and reflection phenomena that caused a reduction in the light penetration depth. When expressed per gram of TiO_2_, the activity of Pt/TiO_2_-PyCF is almost 4-times higher than that of Pt/TiO_2_ (1079 and 273 mmol H_2_/g_TiO2_, respectively), which points to the positive effect of an adequate dispersion of a TiO_2_ phase on a carbonaceous support, forming a highly dispersed and homogeneously distributed titanium dioxide phase. Throughout a 12 h reaction period, the H_2_ production rate progressively decreases, while the CO_2_ production rate increases continuously. This behavior is compatible with an initial period when glycerol dehydrogenation to glyceraldehyde and/or dihydroxyacetone and hydrogen predominates, followed by a period in which comparatively slower C-C cleavage reactions begin to occur, thus generating both H_2_ and CO_2_.

## 1. Introduction

Hydrogen is an energy vector whose combustion only generates water as a by-product. Its production from renewables (green hydrogen) would allow for a reduction in the anthropogenic CO_2_ emissions responsible for global warming. Regarding hydrogen synthetic procedures, the photocatalytic reforming (photoreforming) of biomass-derived oxygenated organic compounds is an interesting alternative for the production of green hydrogen [1].

Although titanium dioxide is the main photocatalyst of choice due to its high stability and low cost [2], there are still some major drawbacks that need to be solved, such as the recombination of photogenerated pairs, fast competitive backward reactions, and the inability to absorb visible light photons. Among the approaches to overcome these disadvantages, the combination or doping of TiO_2_ with noble (e.g., Pt, Au) [3], non-noble (e.g., Cu, Ni) metals [4,5], or non-metal elements (B, C, N, S) [6,7] has been described. This would extend the light absorption range to the visible region and/or the electron–hole pair recombination. Other strategies to enhance titania photocatalytic activity for hydrogen production include the creation of mesoporous titania with high porosity and well-defined channels, which facilitate diffusion or the incorporation of two dimensional materials (such as graphene or transition metal dichalcogenides) or MOF materials, which enhance the separation and transfer of photogenerated charge carriers, among others [8].

The use of a support for semiconductors can contribute to an increase in stability, reusability, or surface area, while the efficient participation of such a support in opto-electrochemical properties is still challenging [9]. Numerous studies have shown that carbon materials can serve as excellent supports and conductive materials for coupling with semiconductors to form hybrid systems for the photocatalytic production of H_2_ [10]. In particular, the formation of composites of titania with carbonaceous materials can result in (i) an increase in the active surface of the semiconductor, (ii) an enhancement in the semiconductor adsorption properties [11], (iii) a photosensitizing effect that decreases the value of the semiconductor bandgap, (iv) an alteration in the defective sites of the heterostructures, and (v) a reduction in the recombination of the generated charges, if the carbons present adequate electronic properties [12,13].

However, using carbon as a support in photocatalytic experiments could have strong light absorption and cause shielding effects, leading to inefficient light absorption by semiconductors [10]. Furthermore, Tolosana-Moranchel et al. presented a study of the optical properties (extinction, absorption, and dispersion coefficients) of different commercial TiO_2_ particulate solids in the photodegradation process of phenol in suspension, showing that these properties depend, to a large extent, on the hydrodynamic particle size (hydrated/solvated particle) and significantly affect the photocatalytic performance of the reaction [14].

Recently, numerous papers have been published that pay special attention to the procedures to be followed in photocatalyzed processes (experimental design and control of reaction parameters) and the most appropriate way to present the results obtained (e.g., photonic efficiency, apparent quantum efficiency, etc.), which allow for drawing relevant conclusions [15,16,17,18,19]. Moreover, when an excessive amount of a photocatalyst is used, most of the photocatalyst cannot absorb sufficient light and the photocatalytic activity is lower when normalized by the unit photocatalyst mass. Therefore, the H_2_ production rate under such conditions cannot reflect the intrinsic performance of the photocatalyst.

Among the non-photonic properties of the photocatalysts that can affect the photocatalytic results are: (i) differences in the crystalline phase of the semiconductor; (ii) differences in the size and shapes of the particles, thereby affecting the extent of light scattered; (iii) differences in the density of surface hydroxyl groups of the semiconductor particle that would affect to the hydrophilic/hydrophobic properties of the solids; (iv) differences in the number and nature of trap sites, both in the lattice and at the surface; and, finally, (v) the adsorption/desorption characteristics of each surface that may vary according to the nature of the photocatalyst material [19]. Thus, the use of carbonaceous materials as TiO_2_ supports could affect the previous properties and, therefore, have a notable influence on the behavior of the final composite.

In previous works, using a commercial mesoporous carbon, it was found that an adequate superficial chemical activation of the carbonaceous material was crucial for an optimal dispersion of the TiO_2_ incorporated through sol–gel. Furthermore, the effect of the incorporation of Pt, as a cocatalyst, to the composite was evaluated for the hydrogen production from aqueous glycerol photoreforming. The absence of water during the sol–gel process ensured the formation of small homogeneously distributed anatase crystals on the activated mesoporous carbon. Hydrogen production for TiO_2_-carbon and Pt/TiO_2_-carbon, expressed as mmol H_2_ per gram of titania, was higher than that achieved with their respective reference materials (pure Evonik P25 and Pt/P25) [20,21].

In this work, with the aim of valorizing this material generated as a by-product in olive mills, two biochars with different porosity and particle size distribution were synthesized from ground olive stones using two different procedures. Subsequently, a TiO_2_ phase was incorporated into these carbons by means of an ultrasound-activated sol–gel procedure and, finally, a Pt metallic phase was photodeposited on this TiO_2_-carbon composite, giving rise to a series of photocatalysts that were thoroughly characterized and tested for the generation of hydrogen by photoreforming the aqueous glycerol solutions. Special attention was paid to the study of operational parameters, such as the catalyst weight or the size of the photocatalyst particles, that could affect the extent of light scattering and shielding, thus hindering the light absorption by the semiconductor active species (TiO_2_).

## 2. Materials and Methods

### 2.1. Materials

TiO_2_ (Evonik, Essen, Germany, P25), titanium (IV) isopropoxide (Sigma-Aldrich, St. Louis, MO, USA, 97%), platinum(IV) chloride solution (Sigma-Aldrich, St. Louis, MO, USA, 8 wt.% in H_2_O), glycerol (Sigma-Aldrich, St. Louis, MO, USA, 99%), 2-propanol (Sigma-Aldrich, St. Louis, MO, USA, >99.8%), methanol (Sigma-Aldrich, St. Louis, MO, USA, 99.9%), mesoporous carbon (Sigma-Aldrich, St. Louis, MO, USA, <500 nm particle size (DLS) >99.95% trace metals basis), HNO_3_ (Sigma-Aldrich, St. Louis, MO, USA, >65%), H_2_SO_4_ (Panreac, Castellar del Vallès, BCN, Spain, 95–98%), AgNO_3_ (Panreac, Castellar del Vallès, BCN, Spain, 99%), and deionized water (resistivity ≥18 MΩ cm, MilliQ Millipore, Billerica, MA, USA) were used in this study.

### 2.2. Synthesis of Carbonaceous Materials, Composites, and Photocatalysts

#### 2.2.1. Synthesis of Biochars from Olive Stones

In this work, two types of biochar were synthesized via carbonization or pyrolysis and the chemical activation of olive stones. The olive stones, supplied by Olivarera de los Pedroches S.C.A., Pozoblanco (Cordoba, Spain), were thoroughly washed with distilled water at room temperature (24 h) in order to remove any remaining pulp, dried at 100 °C (24 h), crushed, and sieved to retain the 1000–3000 µm particle size fraction.

#### 2.2.2. Synthesis of Activated Carbon (AC)

The synthesis of activated carbon (AC) was carried out following a one-step chemical activation method with phosphoric acid, as described by Yakout et al., 2017 [22]. In brief, 50 g of olive stone was impregnated with 200 mL of phosphoric acid solutions (70 wt.%), the mixture being stirred at 85 °C for 4 h. After mixing, 10 g of material was pyrolyzed in a tubular furnace at 600 °C for 2 h (heating rate, 13 °C/min) under nitrogen atmosphere (flow rate, 38 mL/min). After heating, the furnace was cooled down to room temperature under the same nitrogen flow and the solid obtained washed with distilled water until pH 5 in rinse water and then oven-dried at 120 °C for 24 h. The biochar obtained was ground on a ball-type mill at 300 rpm for 15 min and subsequently sieved to retain the particle size fraction between 5 and 74 µm. The solid obtained was labelled as AC (active carbon).

#### 2.2.3. Pyrolyzed Carbon Functionalized (PyCF)

A second type of carbonaceous material was synthesized in two consecutive steps: a) in the first step, a biochar was synthesized via pyrolysis of an olive stone sample according to the procedure reported by Estévez et al., 2020 [23]. Thus, 5 g of olive stones was pyrolyzed in a tubular furnace at 900 °C (heating rate, 10 °C/min) for 1 h under nitrogen atmosphere (flow rate, 50 mL/min). The obtained pyrolysis carbon (PyC) was ground on a ball-type mill at 100 rpm for 3 h and subsequently sieved to retain the 2–70 µm particle size fraction; b) in a second step, the pyrolysis carbon surface was functionalized by treating 1 g of material with 10 mL of a HNO_3_/H_2_SO_4_ mixture (3:1 *v*/*v*) and sonicating the mixture at 25 °C for three hours. Then, the slurry was filtered and washed with deionized water until reaching pH 3 in the rinse water, dried to 120 °C for 6 h, and sieved on a 150 µm mesh, thus leading to the solid labelled as PyCF (pyrolyzed carbon functionalized). More details can be found elsewhere [20].

#### 2.2.4. Mesoporous Carbon Functionalized (MCF)

A third carbonaceous material was obtained via functionalization of a commercial mesoporous carbon. Functionalization was carried out according to a procedure described elsewhere [20]. The carbonaceous material obtained was labelled as MCF (mesoporous carbon functionalized).

#### 2.2.5. Synthesis of the TiO_2_-Biochar Composites

The incorporation of titania at a nominal content of 25 wt.% to the carbons was made through the ultrasonic-assisted sol–gel process using titanium isopropoxide as a precursor in the absence of water following the procedure described in a former work [20,21]. In brief, 1 g of the selected carbon material was added to a solution containing 2.5 mL of titanium tetraisopropoxide in 10 mL of isopropanol. The mixture was sonicated for 4 h at 25 °C and then refluxed at 80 °C for 24 h to achieve the hydrolysis of TiO_2_ on the carbon surface. The resulting solid was filtered, dried at 110 °C for 12 h, and calcined at 400 °C for 2 h (heating rate, 4 °C/min). The TiO_2_-carbon composites, thus obtained, were named TiO_2_-AC, TiO_2_-PyCF, and TiO_2_-MCF for titania supported on active carbon, functionalized pyrolyzed carbon, and functionalized mesoporous carbon, respectively.

#### 2.2.6. Synthesis of Pt/TiO_2_-Carbon Photocatalysts

Platinum was incorporated onto TiO_2_-carbon composites (nominal content of 0.5 wt.%) through the photodeposition method [21]. The chosen TiO_2_-carbon composite was dispersed in a methanol/water (20%, *v*/*v*) solution containing H_2_PtCl_6_ as metallic precursor. Photodeposition was carried out under argon atmosphere by exposing the mixture, under stirring, to the light for 5 h (125 W mercury lamp, λmax = 365 nm, Photochemical Reactors Ltd., Reading, UK). The photocatalyst was filtered, washed, dried overnight, and calcined at 400 °C for 2 h (heating rate, 4 °C/min) in nitrogen atmosphere (flow rate, 50 mL/min). The obtained photocatalysts were named Pt/TiO_2_-AC, Pt/TiO_2_-PyCF, and Pt/TiO_2_-MCF. A scheme on the synthesis can be found as Supplementary Material (Supplementary Scheme S1).

For comparative purposes, an additional photocatalyst was synthesized by incorporating Pt (0.5 wt.%) on commercial titania (Evonik P25) using the same photodeposition procedure described above, thus leading to Pt/TiO_2_ solid.

### 2.3. Characterization Techniques and Procedures

Chemical analysis of the solids was performed via XRF, using a Rigaku ZSX Primus IV spectrometer (Rigaku, Austin, TX, USA). 

Textural properties were determined through N_2_ adsorption–desorption isotherms at boiling temperature on an Autosorb-iQ MP/MP-XR (Quantachrome instruments) (Anton Paar, Graz, Austria). Before measurements, samples were outgassed at 0.1 Pa at 120 °C. 

The analysis through granulometry or particle size distribution (PSD) of the solids was carried out under dynamic light scattering (DLS) on a Zetasizer Nano ZSP (Malvern Panalytical Ltd., Malvern, UK) to analyze solids whose average particle diameter was in a range of 10 to 1000 nm and laser diffraction (LDM) on a Mastersizer 2000 (Malvern Panalytical Ltd., Malvern, UK) for determinations in a range of 0.1 to 1000 microns.

Platinum average particle size was determined from transmission electron microscopy (TEM) images obtained using a JEOL JEM 1400 microscope. Sample was mounted on 3 mm holey carbon copper grids. STEM images were performed on a Thermo Scientific Talos F200i instrument (Thermo Fisher Scientific Inc., Waltham, MA, USA) equipped with a Schottky-type field emission gun (FEG), with an operating voltage of 200 kV, a STEM-HAADF transmission scanning imaging system, and X-ray energy-dispersive microanalysis system (EDX). X-ray diffraction (XRD) patterns were recorded in the 5–80° range on a Bruker D8 Discover A25 diffractometer (Bruker Española S.A., Madrid, Spain) equipped with CuKα radiation, a Ge monochromator, and a Lynxeye detector. Raman spectra were obtained with InVia Raman equipment (Renishaw Ibérica, S.A.U., Barcelona, Spain) furnished with a Leica microscope and a charge coupled device (CCD) detector. Spectra were collected with a green laser (532 nm) in the 100–1800 cm^−1^ Raman shift range, accumulating 20 spectra. IR spectra were obtained on an FT-MIR Bruker Tensor 27 spectrometer at the NIR/MIR unit of the SCAI of University of Cordoba. Diffuse reflectance UV-Vis spectroscopy was performed on a Cary 1E (Agilent, Santa Clara, CA, USA) instrument, using BaSO_4_ as reference material. Surface charge of materials was determined by Z-potential measurement on a Zetasizer Nano ZSP coupled to an MPT-2 autotitrator using NaOH and HCl to modify the pH.

### 2.4. Glycerol Photoreforming Experiments

Glycerol photoreforming was carried out in a 190 cm^3^ cylindrical reactor with the catalyst (0.5 g/L) suspended in a 65 mL solution of glycerol in MilliQ^®^ water (10%, *v*/*v*), which was kept under constant stirring. Both the photocatalytic reaction and Pt photodeposition process were carried out by means of an ultraviolet irradiation source produced by a 125 W medium pressure mercury lamp (Photochemical Reactors Ltd., Reading, UK) that produces predominantly 365–366 nm radiation, with smaller peaks in the ultraviolet and visible region (see Supplementary Figure S1 for additional information). The lamp was cooled throughout the process by passing a flow of thermostated water at 20 ± 1 °C through a jacket. The radiation power emitted by the lamp was measured outside the reactor (see Supplementary Figure S1) with a Newport 841-P-USAB optical power meter, equipped with a Newport 818P Series high-power detector (MKS Instruments, Andover, MA, USA).

The gases produced in the reaction were swept by a flow of Ar (20 mL/min) towards a Hyden HR20 mass spectrometer (Hyden Analytical Ltd., Warrington, UK), where H_2_ and CO_2_ were continuously monitored (*m*/*z* values of 2 and 44, respectively). Since the H_2_ production rate, expressed in µmol/h g_CAT_, depends on the experimental setup and operating conditions [15], additional information on the experimental setup, including information on the reactor and the radiation source, is provided in Supplementary Figure S1. Some experiments were performed in triplicate and the standard deviation for the cumulative production of hydrogen was determined, obtaining an SD value of 7.5%.

## 3. Results and Discussion

### 3.1. Synthesis of Carbonaceous Supports and Photocatalyst

Starting from olive stones, a biochar was obtained by means of a pyrolytic process at 900 °C in a N_2_ atmosphere, obtaining a carbonaceous material that was then activated as a result of acidic treatment (HNO_3_/H_2_SO_4_), giving rise to the PyCF activated carbon. A second carbonaceous material was obtained by impregnating an olive stone sample with H_3_PO_4_ and subsequent heat treatment at 600 °C in a N_2_ atmosphere, leading to the AC solid. Likewise, commercial mesoporous carbon was activated through acid treatment to enhance its acid properties, leading to the functionalized mesoporous carbon, MCF. The subsequent incorporation of a TiO_2_ phase using an ultrasound-activated sol–gel method led to the TiO_2_-carbon solids, on which, finally, platinum was photodeposited in a nominal percentage of 0.5% by weight (Pt/TiO_2_-Carbon). Finally, the commercial TiO_2_ Evonik P25, which is a solid that is commonly used as a reference in photocatalytic processes [24], was incorporated into the experiments both as pure TiO_2_ and as Pt/TiO_2_. All the synthesized solids were characterized from the textural, structural, and chemical point of view using a wide variety of techniques and tested for the hydrogen production from aqueous glycerol photoreforming.

### 3.2. Catalyst Characterization

#### 3.2.1. Textural and Morphological Characterization of Synthesized Solids

**Figure 1:**
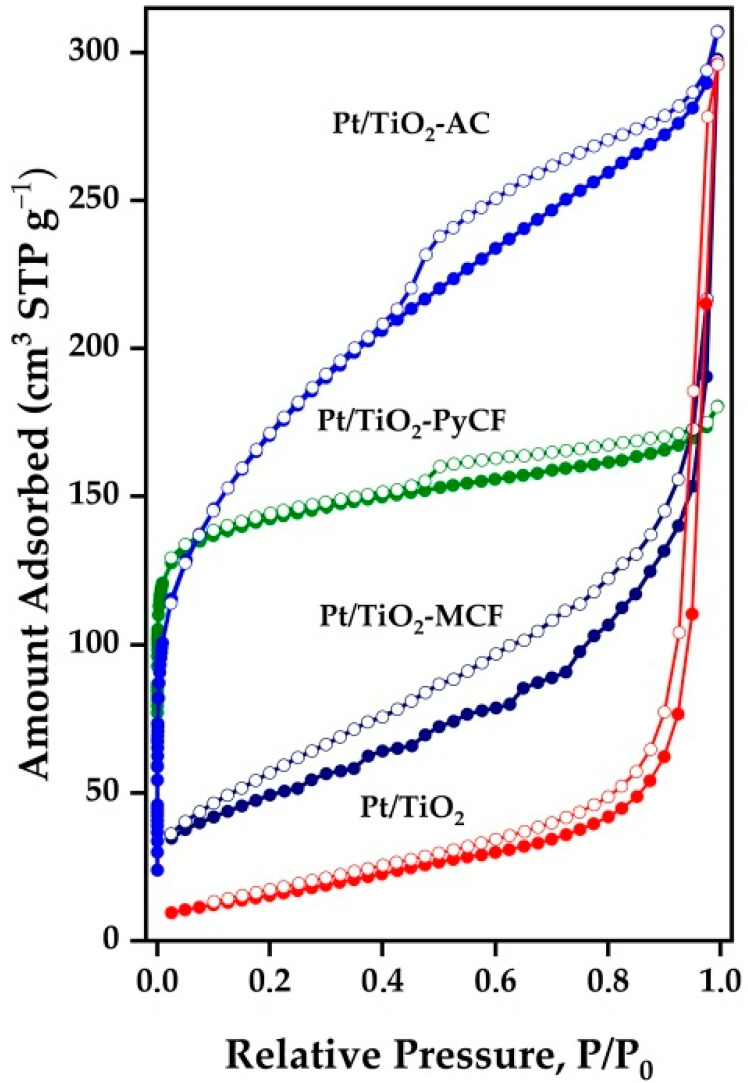
Nitrogen adsorption–desorption isotherms obtained for the synthesized Pt/TiO_2_-carbon photocatalysts.

The textural properties of the solids used in this study were determined by obtaining the nitrogen adsorption–desorption isotherms at their boiling temperature, with the obtained results presented in Table 1 and Figure 1.

The materials obtained from commercial mesoporous carbon (TiO_2_-MCF and Pt/TiO_2_-MCF) presented type IV isotherms according to the IUPAC classification, characteristic of mesoporous materials. These materials developed a monolayer–multilayer adsorption behavior on the mesopore walls, followed by capillary condensation. The hysteresis loop in the adsorption isotherm was H3 type, indicating wedge-shaped pores [25,26]. The TiO_2_-MCF composite had a surface area of 172 m^2^ g^−1^. The incorporation of the metallic phase did not cause any additional change in the textural properties of the Pt/TiO_2_-MCF photocatalyst (S_BET_ 173 m^2^ g^−1^).

The AC biochar-related solids showed an isotherm with mixed characteristics from type I and IV, corresponding to micro- and mesoporous materials, respectively. The hysteresis loop was H4 type, showing a more pronounced adsorption branch at low P/P_0_, which was associated with micropore filling, the pore having a parallel slit shape [22,27]. The TiO_2_-AC material shows a high surface area of 864 m^2^ g^−1^ with mostly mesoporous characteristics (89% volume in mesopores). The subsequent photodeposition of the metallic phase produced a decrease in the surface area to 616 m^2^ g^−1^ for the Pt/TiO_2_-AC catalyst that continued to be a mostly mesoporous solid (82% mesopores), though with 18% of the total volume corresponding to micropores.

On the other hand, the TiO_2_-PyCF composite showed a type II isotherm, associated with non-porous or macroporous materials, according to a plain pyrolyzed material [23]. The surface area of the TiO_2_-PyCF catalyst was 602 m^2^ g^−1^ while the incorporation of the Pt phase led to a specific area of 552 m^2^ g^−1^ for the Pt/TiO_2_-PyCF photocatalyst, a solid with 66% of its pore volume associated with micropores. In the case of Pt/TiO_2_, the solid surface area is also comparable to that of commercial Evonik P25 (63 and 56 m^2^ g ^−1^, respectively).

As for the morphological characterization of the solids, the particle size was determined via dynamic light scattering (DLS) on a Zetasizer Nano ZSP or laser diffraction (LD) on a Mastersizer 2000, depending on the range of the particle size of the measured material, with the results shown in Table 2. The results obtained for the fresh photocatalysts indicated that the largest mean particle size was obtained for the Pt/TiO_2_-PyCF, which showed a volume-weighted mean particle size of ca. 37 µm, while the Pt/TiO_2_-AC solid had an average particle size of 10 µm. On the other hand, the Pt/TiO_2_-MCF photocatalyst, which only contained mesopores, presented the smallest particle size, around 0.3 µm (366 nm). The reference Pt/TiO_2_, which also consisted of mesopores only, showed a similar average particle size of 326 nm. The pronounced differences in the mean particle sizes for the different synthesized photocatalysts could imply different dynamic behavior of the same within the reaction mixtures where they are found, under agitation, at a semiconductor concentration of 0.5 g/L.

In addition, the average particle size was determined for the used (recovered) catalysts, obtaining the results shown in Table 2. Thus, once the photoreforming reaction after (12 h) was finished, the recovered solids showed average particle sizes significantly lower than the fresh ones, with particle size reductions of 56% (Pt/TiO_2_-AC), 54%, (Pt/TiO_2_-PyCF), 30% (Pt/TiO_2_), and 21% (Pt/TiO_2_-MCF). In general, this decrease in the particle size can be attributed to the hydrodynamic behavior caused by the stirring of the photocatalyst in the glycerol/water solution during the experiment [28]. The PSD determination in photocatalysts subjected to stirring in the dark for 12 h indicates that light radiation does not have a significant influence on the particle size reduction (Table 2). This fractionation of the photocatalyst particles during the reaction could affect, in some way, the light penetration into the reaction bulk by changing the scattering phenomena associated with particle size.

**Figure 2:**
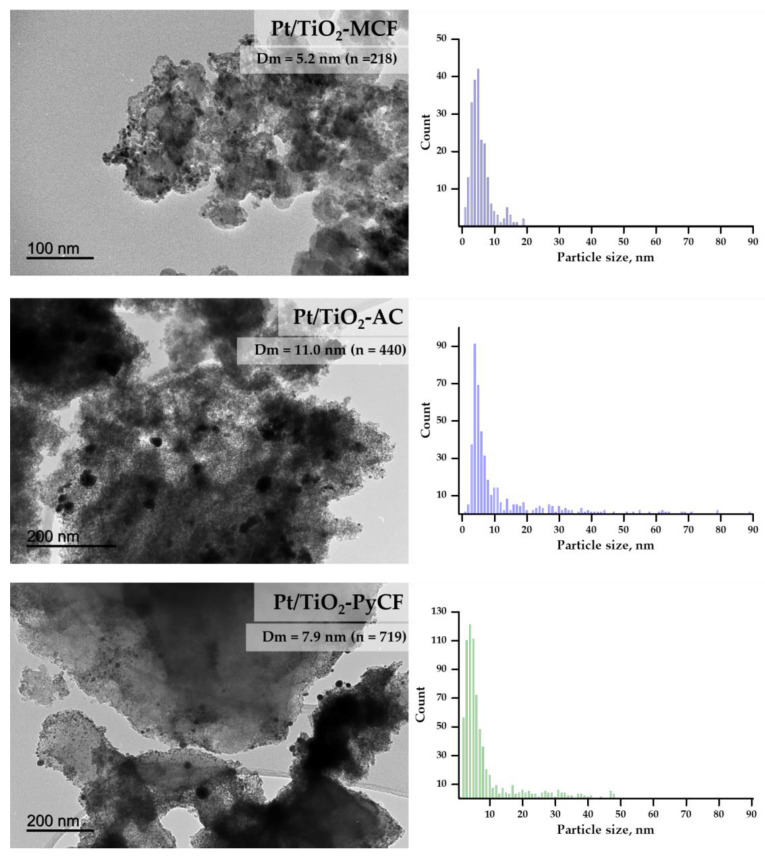
TEM images and Pt particle size distribution histograms obtained for the carbon containing photocatalysts: Pt/TiO_2_-MCF, Pt/TiO_2_-CA, and Pt/TiO_2_-PyCF.

Morphological characterization of the photocatalysts was completed via transmission electron microscopy (TEM), as shown in Figure 2 and Supplementary Figure S2. The Pt mean particle sizes obtained for the Pt/TiO_2_-MCF and Pt/TiO_2_-PyCF solids were 5.2 and 7.9 nm, while the Pt/TiO_2_-AC showed a larger average Pt particle size of about 11.0 nm, with a very heterogeneous distribution of metallic particles. Moreover, low-resolution TEM images (Supplementary Figure S2) confirm the particle sizes obtained via DLS and LD that fall in the nanometric scale for the Pt/TiO_2_-MCF solid and on the micron scale for the Pt/TiO_2_-AC and Pt/TiO_2_-PyCF (Table 2).

#### 3.2.2. Chemical and Structural Characterization of Carbonaceous Materials Synthesized

The chemical composition of the synthesized solids was analyzed via X-ray fluorescence as well as STEM-EDX, with the results obtained shown in Table 3 and in Supplementary Figures S3–S5, respectively.

During the synthesis of biochar from olive stones, the use of pyrolysis conditions increased the yield of carbonaceous material since the anaerobic conditions during the high-temperature treatment decreased the combustion and so the amount of ashes [23]. The XRF chemical analyses of the washed carbonaceous supports yielded between 97 and 99% by weight as carbon: 96.0% for AC, 99.7% for PyCF, and 99.6% for MCF. Moreover, for the AC, the chemical analysis gave significant amounts of phosphorus (ca. 3% by weight) associated with the presence of phosphorous functional groups formed from the H_3_PO_4_ used in the chemical activation process. In addition, trace elements, such as sulfur, silicon, aluminum, iron, phosphorus, calcium, sodium, potassium, and titanium, were found (overall, less than 1% by weight).

Regarding the amount of titania incorporated on the carbonaceous support, the XRF results showed that the amount of TiO_2_ incorporated differs considerably between solids, being 8.3% for TiO_2_-PyCF, 29% for Pt/TiO_2_-MCF, and 41.4% for Pt/TiO_2_-AC (Table 3). These results indicate that the efficiency of the TiO_2_ sol–gel incorporation process on carbonaceous supports depends, to a large extent, on the physicochemical properties of the biochar used, such as total pore volume (Table 1), particle size (Table 2), or surface isoelectric point (Table 3), among others. Thus, for the MCF, a mesoporous solid with a fairly homogeneous particle size of around 366 nm, the incorporated amount of TiO_2_ is quite close to the nominal value (25% by weight). On the other hand, for the biochars obtained from olive stones, AC and PyCF, the larger size and heterogeneity of the carbon particles led to a very irregular incorporation of the titania phase, the lower TiO_2_ incorporation being for the PyCF, the solid with the lowest total pore volume, as reported in Table 1. As for the Pt content, the metal loading was 0.49%, 0.69%, and 0.83% by weight for the Pt/TiO_2_-PyCF, Pt/TiO_2_-AC, and Pt/TiO_2_-MCF, respectively (Table 3).

In order to gain a deeper insight into the spatial distribution of the different chemical constituents of the photocatalysts, analysis of the solids using scanning transmission electron microscopy (STEM) with chemical mapping using energy-dispersive X-ray spectroscopy (EDS) was carried out, and the results are presented in Supplementary Figures S3–S5. STEM photographs show that titania is dispersed over practically the entire surface of the carbonaceous support, thus confirming that the sol–gel procedure used for titania incorporation is adequate to maximize the exposed surface of TiO_2_. Furthermore, since the incorporation of platinum was carried out by means of a photodeposition procedure and the main photoactive material in the composites is titania, the Pt particles were mostly located over the TiO_2_ phase, as confirmed by EDS mapping. 

**Figure 3:**
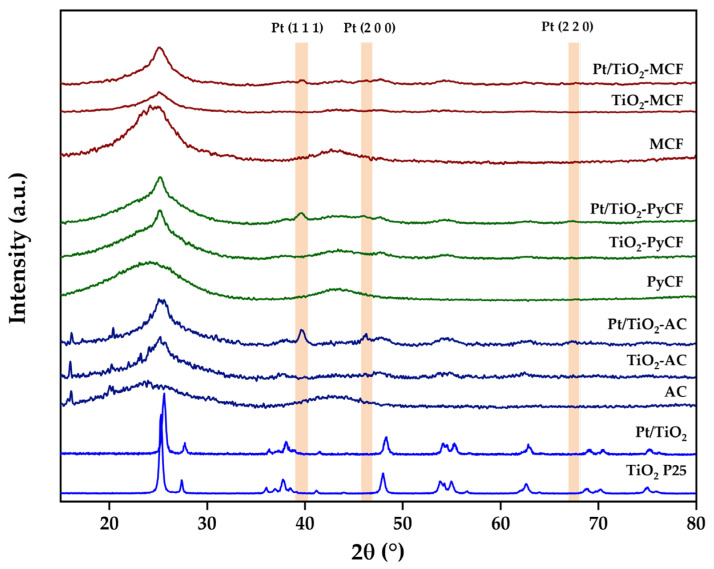
X-ray diffraction patterns obtained for the carbons, the TiO_2_-carbon composites and Pt/TiO2-carbon photocatalysts synthesized in this work.

The microstructural characterization of the synthesized solids was carried out by means of X-ray diffraction and Raman spectroscopy. Figure 3 shows the diffractograms obtained for the synthesized carbons, the TiO_2_-carbon composites, and the Pt/TiO_2_-carbon photocatalysts. The carbonaceous materials showed typical diffractograms of amorphous materials with broad and relatively low-intensity reflections at 23° (002) and 43° (100), indicating the degree of carbon layer stacking and the degree of ordered hexagonal structures, respectively [29]. It is worth noting that the diffractograms obtained for the AC biochar and related solids present some narrow and low-intensity diffraction bands at 2θ values between 16° and 20° that could be associated with the inorganic matter typical of the ashes from the biomass activation method, such as calcite (CaCO_3_), opal (SiO_2_•nH_2_O), quartz (SiO_2_), weddellite (Ca(CO_2_)_2_•(H_2_O)_2_), whewellite (Ca(CO_2_)_2_•H_2_O), portlandite (Ca(OH)_2_), or whitlockite (Ca_3_(PO_4_)_2_), among others [30]. The incorporation of the TiO_2_ phase on the carbons does not lead to clear diffraction bands in the composites, but anatase incipient crystallization bands could be observed.

As for the Pt phase, the diffractograms of the photocatalysts showed a typical signal at 2θ = 39.47° associated with the (111) reflection of Pt metal (JCPDS 4–802) [31], which is clearer for Pt/TiO_2_-AC and, to a lesser extent, for Pt/TiO_2_-PyCF. As for the Pt/TiO_2_-MCF, although the signals associated with metallic Pt were also observed, they were very weak, which indicates smaller crystallite size. For a Pt content as low as 0.5%, the signals associated with Pt were observed, indicating that Pt crystallites are large, especially for the olive-stone-derived solids, Pt/TiO_2_-AC and Pt/TiO_2_-PyCF, which is consistent with the conclusions reached from TEM measurements. 

Regarding the Pt/TiO_2_ catalyst, its diffractogram exhibits the anatase and rutile diffraction bands corresponding to its composition (ca. 80% anatase and 20% rutile) (Anatase, JCPDS card Nr. 21-1272) (Rutile, JCPDS Card Nr. 21-1276) [32,33]. No diffraction bands associated with Pt are observed, probably due to a good metal dispersion with small Pt crystallites.

**Figure 4:**
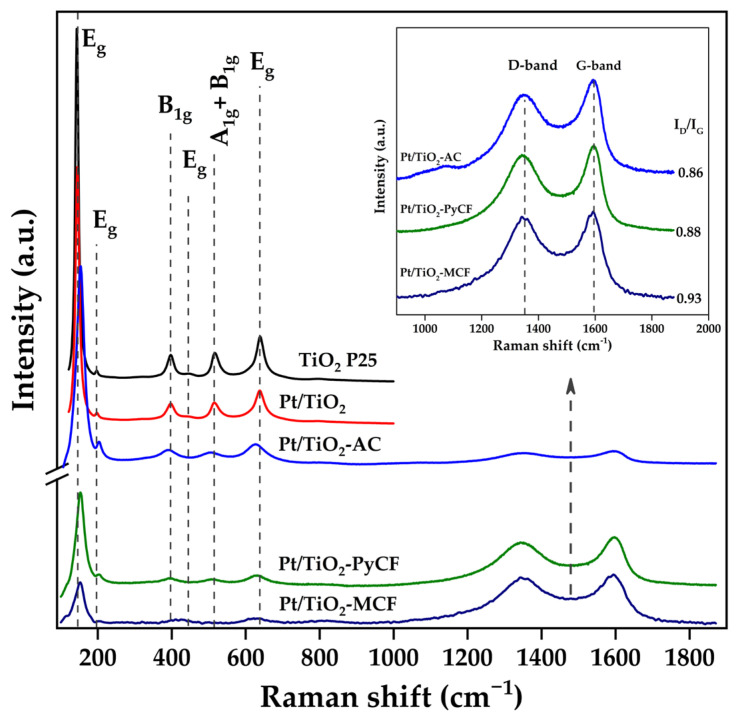
Raman spectra obtained for the studied photocatalysts. The insert shows the detail of the zone of appearance of the D and G bands of the carbons.

Figure 4 shows the Raman spectra obtained for the synthesized solids. Raman spectroscopy is a very sensitive technique to analyze the different crystalline phases of a titanium oxide sample. Thus, for the TiO_2_-carbon composites, several signals associated with the titania can be distinguished at low Raman shift, the most intense ones being those observed at 149 cm^−1^ (E_g_ vibration mode), 396 cm^−1^ (A_1g_), 515 cm^−1^ (A_1g_ + B_1g_), and 636 cm^−1^ (E_g_), which are typical signals for the anatase phase of TiO_2_ [34,35]. No signals were observed that could be associated with other TiO_2_ phases, such as rutile or brookite.

Furthermore, Raman could also give valuable information for the carbonaceous materials obtained from signals appearing at high Raman shift [36]. The two principal signals are the D and G bands at around 1352 cm^−1^ and 1600 cm^−1^, respectively. The D band (A_1g_ Raman active mode) is associated with disordered (amorphous) carbon structures, while the G band (E_2g_ vibration mode) is associated with a hexagonal carbon structure. Therefore, the ID/IG intensity ratio can be used to obtain an estimation of the change in structure and the order of crystals of the carbonaceous support: the higher this ratio, the higher the defect content in the examined carbon [29,37]. The values of the ID/IG ratios obtained for the carbon-containing photocatalysts synthesized are 0.88, 0.86, and 0.93 for the Pt/TiO_2_-PyCF, Pt/TiO_2_-AC, and Pt/TiO_2_-MCF, respectively. The lower values obtained for Pt/TiO_2_-AC and Pt/TiO_2_-PyCF, as compared to Pt/TiO_2_-MCF, indicate a larger proportion of defects.

#### 3.2.3. Optoelectronic and Electric Characterization of the Solids

To determine the optoelectronic properties of carbons, TiO_2_-carbon composites, and Pt/TiO_2_-carbon photocatalysts, the UV-Vis absorption spectra were obtained for all the solids synthesized together with commercial Evonik P25 and Pt/TiO_2_ (Supplementary Figure S6). For the Evonik P25, the UV-Vis spectra showed intense absorption below 400 nm due to the ligand-to-metal charge transfer (LMCT) associated with the O_2_^−^ to Ti^+4^ transitions. The band gap obtained for Evonik P25 from the Tauc’s plot (indirect transition model) was 3.14 eV. Synthesized Pt/TiO_2_ presented similar behavior to the support alone. As for the carbon materials, MCF, AC, and PyCF present a continuous absorption profile throughout the entire recorded interval, which extends from the UV to the visible spectrum region. The incorporation of the TiO_2_ phase on the carbons and the subsequent incorporation of the Pt metallic phase do not change the characteristics of the absorption profile recorded for the photocatalysts (Supplementary Figure S6). 

The isoelectric point (IEP) of the solids was obtained by determining the pH value where the zeta potential was zero. The measurement of the IEP can give us some hints on the modifications in surface groups occurring on the initial mesoporous carbon material with the different synthetic treatments. Supplementary Figure S7 presents the evolution of zeta potential with pH value for the different solids used in the present work, and Table 3 shows the isoelectric point (IEP) determined for the Pt/TiO_2_-carbon photocatalysts synthesized in this work. Regarding the carbonaceous materials, the isoelectric point obtained for AC, PyCF, and MCF was 2.2, 2.8, and 4.4, respectively. The incorporation of the TiO_2_ phase on the carbonaceous supports produces an increase in the IEP for the composites up to pH values of 6.1, 4.3, and 5.4 for TiO_2_-AC, TiO_2_-PyCF, and TiO_2_-MCF, respectively. This may be associated with the increase in the surface of titanol groups at the expense of acidic groups on the biochar’s surface. Finally, the photodeposition of Pt on the composite produces only slight changes in the IEP of the photocatalysts, leading to 5.9 for Pt/TiO_2_-AC, 5.7 for Pt/TiO_2_-PyCF, and 4.5 for Pt/TiO_2_-MCF. The isoelectric point obtained for the Pt/TiO_2_ catalyst was 7.5 (Table 3).

All in all, three tri-component solids made up of Pt, TiO_2_, and carbon were synthesized. The carbons, used as support, transfer, to a large extent, their physical and chemical properties to the final photocatalysts. The solid Pt/TiO_2_-MCF presents carbon particles in the nanometric range and mesoporous characteristics, with a surface area of 173 m^2^ g^−1^. It is made up of 29% by weight of TiO_2_ and a Pt loading of 0.83%. On the other hand, the Pt/TiO_2_-AC was made up of carbon particles of about 10 microns and had mainly mesoporous characteristics, although with 18% micropores (S_BET_ 616 m^2^ g^−1^). Its TiO_2_ content was high (41% by weight) and its Pt charge was 0.69%. Finally, the Pt/TiO_2_-PyCF catalyst was made up of large carbon particles (36 microns) and was mostly microporous (66% micropore volume), with a BET surface area of 552 m^2^ g^−1^. Its TiO_2_ content was low (8% by weight), according to its low total pore volume, and its Pt content was 0.44%.

### 3.3. Glycerol Photoreforming

The synthesized catalysts, consisting of a photoactive semiconductor (TiO_2_), a cocatalyst (Pt), and a carbonaceous carrier material, which, in principle, acts as a mere support, were tested for hydrogen production from aqueous glycerol photoreforming, whose overall reaction, considering pure photoreforming, is shown in Equation (1):

C_3_H_8_O_3_ + 3H_2_O → 3CO_2_ + 7H_2_ (1)

The preliminary tests performed showed that solids without TiO_2_ in their composition were not active for hydrogen production, indicating that the process was photocatalytic and that TiO_2_, as an active semiconductor, was required. Furthermore, only TiO_2_-based solids with Pt as a cocatalyst gave remarkable hydrogen production. Therefore, the improvement observed in the hydrogen production was due to a synergistic effect between a well-dispersed TiO_2_ phase on the carbonaceous support and the incorporation of the Pt phase as a co-catalyst.

However, it is possible that biochars used as carriers positively affect the photocatalytic behavior of the semiconductor, for example, by slowing down the rate of electron–hole recombination, thus going beyond their function as a mere support. On the contrary, black carbonaceous supports would have strong light absorption that could lead to shielding effects and inefficient light absorption by TiO_2_. Taking this into account, in this work, the photocatalyzed hydrogen production is expressed both as hydrogen produced per gram of catalyst (µmol H_2_/g_CAT_) and per gram of TiO_2_ (µmol H_2_/g_TiO2_).

Photocatalytic hydrogen production depends on many parameters, from reactor type and geometry to light intensity and wavelength. There is a basic premise for a heterogeneously photocatalyzed reaction to take place efficiently, which is that a majority of the semiconductor particles are accessible in the light radiation used in the process. Once this premise is fulfilled, the ability of the semiconductor to create the electronic carriers (e_CB_^−^/h_VB_^+^) and the efficiency of these to produce a chemical reaction will determine the final activity of the process. From a practical point of view, this accessibility of light to semiconductor particles can be negatively affected by factors such as high opacity in the reaction medium, which would prevent the effective transmission of light. At this point, the use of dark (black) photocatalysts should be questioned, since, to a greater or lesser extent, they can shield the radiation emitted from the lamp. Moreover, it would be necessary to evaluate the ability to keep semiconductor particles in a suitable suspension, thus allowing for their interaction with photons. The stirring speed and the average semiconductor particle size will be relevant parameters to achieve a good suspension of the photocatalyst.

**Figure 5:**
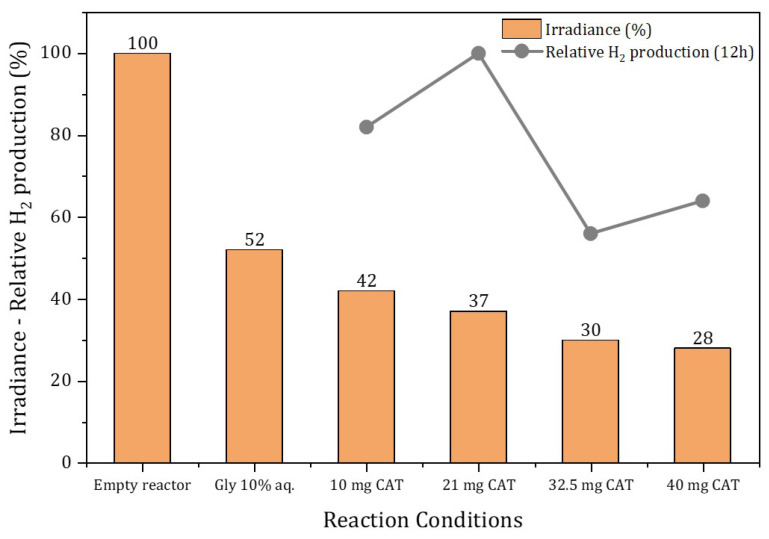
Irradiance externally measured as a function of different reaction conditions. The 100% of irradiance corresponds to 5643 W/m^2^ measured for the empty reactor (only with lamp water cooling). In the ‘Gly 10%aq.’ experiment, no catalyst was present. For the experiments with different catalyst amounts, a ‘Gly 10%aq.’ solution with the Pt/TiO_2_-AC weight indicated was used. See Supplementary Figure S1 for additional details of photoreactor geometry.

To gain further insight into this point, we focused the study on the Pt/TiO_2_-AC catalyst, which, despite being mostly mesoporous, had a high mean particle size (10.4 µm) that can affect both the hydrodynamic behavior and opacity of the reaction mixture. Thus, the radiation that transversely passed through the entire photoreactor was measured, from the inner lamp through both the cooling water and the reaction medium to the outside of the reactor, where an optical power meter was placed, with the results shown in Figure 5 (see Supplementary Figure S1 for additional details of photoreactor geometry).

According to Figure 5, the radiation that passes through the empty system, with only the lamp cooling water, is 5463 W/m^2^. However, when the 10% aqueous glycerol solution is introduced into the photoreactor, without a catalyst, the outgoing radiation drops to 52% (2936 W/m^2^). In addition to the optical power measured, Figure 5 also shows the relative hydrogen production obtained after 12 h of reaction with different amounts of Pt/TiO_2_-AC in the reaction medium, in the 10–40 mg range (0.15–0.62 g/L). This figure suggests that there are two opposite effects that affect the activity. Thus, the increase in the amount of catalyst leads to an increase in the production of hydrogen, obtaining a maximum of 20 mg of the catalyst, from which the activity drops. This drop, quite abrupt, could be associated with an increase in the opacity of the solution at high catalyst loadings, as confirmed by measuring the radiation coming out of the reactor under these conditions. This figure revealed that the introduction of progressive amounts of photocatalysts in the slurry produced an additional drop in the outcoming radiation. Thus, for a catalyst concentration of 0.5 g/L, the outcoming radiation dropped down to 30% (1714 W/m^2^) and, from this value up, although the solution’s opacity continued to increase, the outgoing radiation remained practically constant (0.62 g/L, 28% outcoming radiation). This could be explained because the H_2_ evolution rate is not necessarily proportional to the mass of the photocatalyst used, since incident light also depends on the concentration and dispersion of the photocatalyst, provoking limitations in light absorption and diffusion on the slurry. Thus, at high photocatalyst loading, hydrogen production will decrease because of reduced penetration depth and light scattering and reflection phenomena [15].

**Figure 6:**
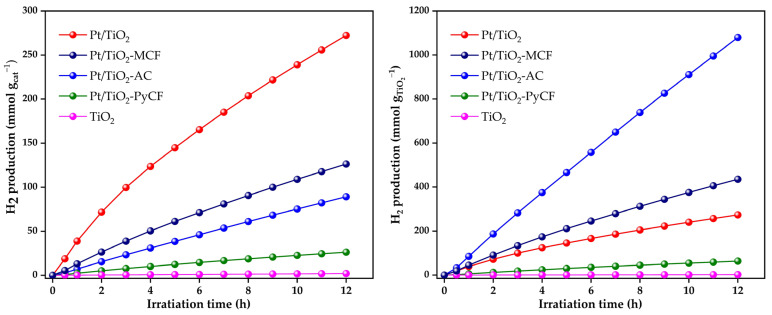
Accumulated hydrogen production per gram of catalyst (**left**) and per gram of TiO_2_ (**right**) obtained for the solids tested in this work. Reactions were carried out under Ar flow (20 mL/min) with 65 mL of a 10% glycerol aqueous solution, a catalyst loading of 0.5 g/L and reaction temperature of 20 °C.

Figure 6 shows the cumulative hydrogen production for the synthesized solids for a reaction period of 12 h. Pure Evonik P25 was introduced for comparison. In all cases, the H_2_/CO_2_ ratio was higher than the stoichiometric value considering pure photoreforming, which is supportive of the generation of intermediates. Regarding the cumulative hydrogen production per gram of catalyst (Figure 6 left), Pt/TiO_2_ was the most active catalyst. Among the catalysts that contain carbonaceous materials in their composition, the Pt/TiO_2_-MCF catalyst presents the best cumulative H_2_ production with 126 mmol H_2_/g_CAT_. According to the characterization results shown above, this photocatalyst is a mesoporous solid with a mean particle size of 366 nm (Table 1 and Table 2). Next, in hydrogen production, we obtained the Pt/TiO_2_-AC solid with 89 mmol H_2_/g_CAT_, which was a mostly mesoporous solid but with a larger mean particle size (10.4 µm, Table 2). Finally, the least active photocatalyst was the Pt/TiO_2_-PyCF catalyst (26 mmol H_2_/g_CAT_), which was a mostly microporous solid with the largest particle size among those synthesized (36.7 µm, Table 2). It is relevant to indicate that the two catalysts that perform best in hydrogen production are those with the smallest particle size (with only a few hundred nanometers). On the other hand, the Pt/TiO_2_-AC and Pt/TiO_2_-PyCF catalysts, with particle sizes of several tens of microns, are the ones that exhibit the worst behavior in terms of hydrogen production. Thus, the results seem to indicate that a mesoporous nature of the photocatalyst leads to better H_2_ production results, while, on the other hand, a smaller particle size seems to be a favorable factor.

Considering the differences in TiO_2_ loading on the solids and to evaluate the intrinsic activity associated with TiO_2_ and determine the possible effects of the carbonaceous material used as a support, Figure 6 (right) shows the results expressed as mmol of H_2_ per gram of TiO_2_. These results show that the most active catalyst was Pt/TiO_2_-PyCF, which had only an 8% TiO_2_ in its composition, followed by Pt/TiO_2_-MCF (29% TiO_2_) and, finally, Pt/TiO_2_-AC (41% TiO_2_). Comparatively, the activity of Pt/TiO_2_-PyCF per gram of TiO_2_ was almost 4-times higher than that of Pt/TiO_2_ (1079 and 273 mmol H_2_/g_TiO2_, respectively), which points to the positive effect of an adequate dispersion of a TiO_2_ phase on a carbonaceous support, forming a highly dispersed and homogeneously distributed titanium dioxide phase.

Since the particle size of the catalyst seems to be an important parameter when establishing the reaction conditions in photocatalyzed processes, a prospective study was undertaken for the synthesized catalysts. Thus, in addition to the average particle size of the as-synthesized catalysts, changes in their particle sizes during the reaction were studied since they may affect the long-time development of the photocatalyzed process (Table 2 and Supplementary Material S8). 

Table 2 shows that the catalysts recovered after 12 h of reaction generally showed a reduction in the average particle size, much more evident for those solids with a larger initial particle size, Pt/TiO_2_-AC and Pt/TiO_2_-PyCF catalysts, for which the reduction in the average particle size was 57 and 54%, respectively. Both are solids with some microporosity, which also had the largest mean particle sizes before the reaction. On the other hand, the observed particle size reductions are smaller for the Pt/TiO_2_ (30% reduction) and Pt/TiO_2_-MCF (21% reduction) catalysts, with both being fundamentally mesoporous catalysts and having the smallest particle sizes before the reaction. The reasons for the observed drop in particle sizes could be due to the stirring process to which the reaction medium was subjected [38], although the incident radiation could also be responsible for a part of the fragmentation of the particles. Thus, Table 2 shows that when a reaction mixture is shaken in the dark, the particle size drops to a lesser extent than when it is shaken and illuminated simultaneously.

Since the Pt/TiO_2_-PyCF and Pt/TiO_2_-AC catalysts were those undergoing greater changes in average particle size during the reaction, a deeper study of their behavior during the reaction was carried out. In Supplementary Figure S9, the H_2_ and CO_2_ evolution rate during the whole 12 h reaction period is presented. It is observed that, after 12 h of reaction, the hydrogen evolution rate suffers a decrease of approximately 23% for the Pt/TiO_2_-AC catalyst and a 39% drop for the Pt/TiO_2_-PyCF. In both cases, this fall is more intense initially, although it is maintained throughout the entire period. On the contrary, as for the CO_2_ evolution, there is a continuous increase in the rate of CO_2_ production throughout the 12 h reaction, such that the final CO_2_ production rate is 2.5-times higher than the initial for the Pt/TiO_2_-AC catalyst and almost 8-times higher for the Pt/TiO_2_-PyCF. Sandwald et al. reported that the H_2_ evolution rates declined by 34% after 12 h of reaction and related this drop to a first-order dependence on glycerol concentration (initial glycerol concentration of 0.01 mM; conversion after 12 h of reaction, 39%) [39]. However, in our experiments, the glycerol concentration was 140-times higher (1.4 M) and glycerol conversion after 12 h was below 1%. Therefore, the drop in the rate of hydrogen production during the reaction may be due to another factor inherent to the reaction itself.

The evolution of the H_2_/CO_2_ ratio can provide some additional information in this regard. In this sense, we must remember that the H_2_/CO_2_ ratio corresponding to pure photoreforming is 2.3 (Equation (1)). For Pt/TiO_2_-AC, the initial H_2_/CO_2_ ratio is 18, while after 12 h of reaction, it drops to 5.5. Regarding Pt/TiO_2_-PyCF, the initial ratio is higher than 80, dropping after 12 h of reaction to a value of 7.0. These results seem to indicate that glycerol that is initially adsorbed on the photocatalyst is easily oxidized to glyceraldehyde (or dihydroxyacetone) through a process, which does not involve breaking C-C bonds but the consumption of two h_VB_^+^, which enables the use of two e_CB_^−^ for two protons reducing to H_2_. Thus, a high amount of hydrogen is formed at the initial stages of the reaction, being associated with a high H_2_/CO_2_ ratio. However, these initially formed intermediates undergo progressive C-C bond cleavage reactions, leading to the release of CO_2_ that is reflected in the progressive increase in carbon dioxide evolving as the reaction progresses [39]. The progressive drop in the H_2_ formation as the reaction proceeds indicates that the rate of the initial dehydrogenation process is higher than that of C-C bond breaking that takes place as the photoreforming process advances. As a result, the H_2_/CO_2_ ratio decreases until approaching the theoretical one (2.3).

**Figure 7:**
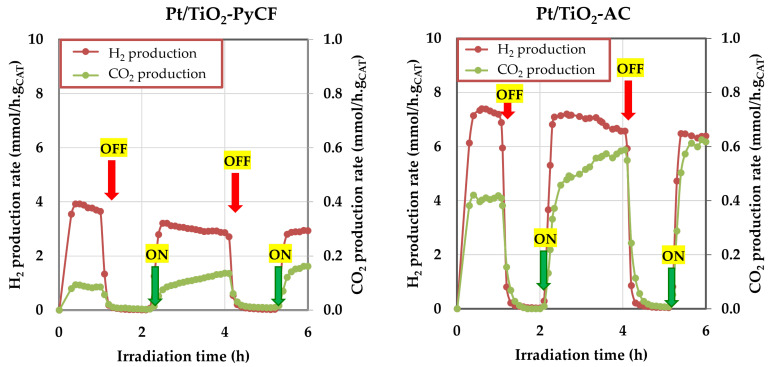
On/Off experiments performed during the glycerol photocatalytic reforming over the Pt/TiO_2_-PyCF and Pt/TiO_2_-AC catalysts. Reactions were carried out under Ar flow (20 mL/min) with 65 mL of a 10% glycerol aqueous solution, a catalyst loading of 0.5 g/L, and reaction temperature of 20 °C.

Therefore, the above scenario cannot be associated with a deactivation process, or at least not with an obvious one, since although the rate of hydrogen production falls, the rate of CO_2_ production increases significantly, especially for the Pt/TiO_2_-PyCF catalyst. To deepen this study, some additional experiments were carried out, consisting of turning off/on the lamp during first stages of the reaction and analyzing the behavior of the H_2_ and CO_2_ production rates to assess whether the changes in reaction rate are reversible or not (Figure 7). When the lamp is turned off, both the sweeping gas flow and the agitation remained unchanged, while the m/z signals corresponding to H_2_ and CO_2_ evolved and continued to be monitored. The results indicate that, after turning off the lamp, the production of both hydrogen and CO_2_ dropped to zero in a few minutes. When, one hour later, the light was turned on again, the production of both gases recovered to the level prior to switching off the lamp. The initial production level of the reaction was not reached, although the observed trends of falling in the rate of H_2_ production and progressive increase in the rate of CO_2_ production were restored, thus assuming that, after one hour of stirring in the dark, the reaction resumes at the exact point where the light was turned off.

Considering all of the above information together, it seems that the drop in the rate of hydrogen production and the parallel increase in the CO_2_ are neither due to kinetic reasons (glycerol conversion after 12 h is less than 1%) nor to a clear catalyst deactivation process but to the fact that the first steps of the reaction that lead to glyceraldehyde or dihydroxyacetone and generate H_2_ (equivalent to a dehydrogenation process) occur easily, thus generating a large amount of hydrogen in the effluent [39]. Subsequently, these products, still adsorbed on the catalyst surface, undergo C-C cleavage reactions that form both CO_2_ and H2, thus justifying the progressive increase in the produced amount of CO_2_. However, these C-C cleavage reactions must be slower, which justifies the progressive decrease in the H_2_ production rate.

## 4. Conclusions

Three photocatalysts made up of Pt as a co-catalyst, TiO_2_ as an active semiconductor, and carbon as a support were synthesized, characterized, and tested for photocatalytic hydrogen production from aqueous glycerol photoreforming. The solid Pt/TiO_2_-MCF exhibited carbon nanoparticles and mesoporous characteristics, with relatively low surface area and 29% by weight of TiO_2_. On the other hand, the Pt/TiO_2_-AC, was made up of olive-stone-derived carbon particles of about 10 microns, with mainly mesoporous characteristics (18% micropores), high surface area, and high TiO_2_ content (41% by weight). Finally, the Pt/TiO_2_-PyCF catalyst was made up of large carbon particles (36 microns), being mostly microporous with relatively high surface area and low TiO_2_ content (8% by weight). The carbons used as a support transfer, to a large extent, their physical and chemical properties to the final photocatalysts.

The study of the operating parameters in glycerol photoreforming showed that the H_2_ evolution rate is not necessarily proportional to the mass of the photocatalyst used, since incident light also depends on the concentration and dispersion of the photocatalyst, causing limitations in light absorption and diffusion on the slurry. Thus, at high photocatalyst loading, hydrogen production decreased, probably because of a reduced penetration depth and light scattering and reflection phenomena.

The results obtained per gram of catalyst indicate that a mesoporous nature of the photocatalyst leads to better H_2_ production results, while a smaller particle size also seems to be a favorable factor. Moreover, the results per gram of TiO_2_ revealed that the activity of Pt/TiO_2_-PyCF is almost 4-times more active than Pt/TiO_2_ (1079 and 273 mmol H_2_/g_TiO2_, respectively), which points to the positive effect of an adequate dispersion of a TiO_2_ phase on a carbonaceous support.

The study of the photoreforming process for 12 h of reaction showed a progressive decrease in the amount of hydrogen generated, while an increase in CO_2_ was observed. These results are neither due to kinetic reasons nor to a catalyst deactivation process but to a rapid initial dehydrogenation process to glyceraldehyde and dihydroxyacetone, followed by subsequent C-C cleavage processes that take place at a slower speed.

## Figures and Tables

**Table 1:** Textural properties of the different composites and photocatalysts synthesized in this work.

Sample	^a^ S_BET_ (m^2^/g)	^b^ V_total_ (10^−2^ cm^3^/g)	^c^ V_mic_ (10^−2^ cm^3^/g)	Average Pore Size (nm)	Micropore Volume (%)	Mesopore Volume (%)
Pt/TiO_2_-MCF	173	46	0	11.0	0	100
TiO_2_-MCF	172	115	0	26.8	0	100
Pt/TiO_2_-AC	616	48	8	3.1	18	82
TiO_2_-AC	864	71	7	3.3	11	89
Pt/TiO_2_-PyCF	552	28	18	2.1	66	34
TiO_2_-PyCF	602	28	23	1.5	85	15
Pt/TiO_2_ P25	63	46	0	29.0	0	100
TiO_2_ P25	56	65	0	45.9	0	100

^a^ Specific surface area (BET); ^b^ total volume (BET); ^c^ micropore volume (De Boer method).

**Table 2:** Average particle sizes measured in different conditions for the solids synthesized in this work as determined by dynamic light scattering (DLS) and laser diffraction depending on the range of particle size analyzed.

Catalyst	Conditions	^a^ Z-Ave (nm)	^b^ D (4,3) Vol. Weighed Mean (µm)
Pt/TiO_2_-MCF	As synthesized Recovered after use (12 h) After stirring without light (12 h)	366 ± 16 290 ± 18 297 ± 4	
Pt/TiO_2_-AC	As synthesized Recovered after use (12 h) After stirring without light (12 h)		10.41 ± 1.14 4.54 ± 0.04 6.53 ± 0.37
Pt/TiO_2_-PyCF	As synthesized Recovered after use (12 h) After stirring without light (12 h)		36.68 ± 3.03 16.79 ± 0.50 20.29 ± 1.98
Pt/TiO_2_	As synthesized Recovered after use (12 h) After stirring without light (12 h)	326 ± 23 229 ± 10 -	

^a^ Average particle size as determined by dynamic light scattering (DLS) measurement on a Zetasizer Nano ZSP. ^b^ Volume weighed mean as determined by measured using a laser particle sizer Mastersizer 2000.

**Table 3:** Chemical composition (XRF), Pt particle size (TEM), and isoelectric point for the synthesized photocatalysts.

Catalyst	^a^ Pt (wt.%)	^a^ TiO_2_ (wt.%)	Pt Particle Size (TEM, nm)	^b^ pH_IEP_
Pt/TiO_2_-MCF	0.83	29.0	5.2	4.5
Pt/TiO_2_-AC	0.69	41.4	11.0	5.9
Pt/TiO_2_-PyCF	0.49	8.3	7.9	5.7
Pt/TiO_2_	0.44	99.5	4.4	7.4

^a^ Platinum and TiO_2_ content by XRF; ^b^ isoelectric point (IEP).

## Data Availability

Data will be available on request.

## Supplementary Materials

The following supporting information can be downloaded at: https://www.mdpi.com/article/10.3390/nano13091511/s1, Scheme S1: Schematic illustration of the synthesis of the catalysts; Figure S1: Experimental setup for the aqueous glycerol photoreforming experiments; Figure S2: Morphological aspect of the synthesized photocatalysts from low-resolution transmission electron microscopy images. Pt/TiO_2_-AC (left), Pt/TiO_2_-PyCF (center), and Pt/TiO_2_-MCF (right); Figure S3: TEM/STEM and EDS images obtained for the Pt/TiO_2_-MCF solid; Figure S4: TEM/STEM and EDS images obtained for the Pt/TiO_2_-AC solid; Figure S5: TEM/STEM and EDS images obtained for the Pt/TiO_2_-PyCF solid; Figure S6: UV-vis spectra obtained for the Pt/TiO_2_-carbon photocatalysts prepared in this work; Figure S7: Isoelectric point (IEP) plot for the carbon materials, TiO_2_-carbon composites and the Pt/TiO_2_-carbon photocatalysts prepared in this work. TiO_2_ Evonik P25 and Pt/TiO_2_ were also included for comparison; Figure S8: Normalized particle size distribution (intensity and volume) of the photocatalysts. DLS studies based on intensity–volume for Pt/TiO_2_ (a,A) and Pt/TiO_2_-MCF (b,B). LDA studies based on volume for Pt-TiO_2_-AC (c) and Pt/TiO_2_-PyCF (d); Figure S9: Hydrogen and carbon dioxide evolution during the glycerol photoreforming over Pt/TiO_2_-PyCF and Pt/TiO_2_-AC photocatalysts. Reactions were carried out under Ar flow (20 mL/min) with 65 mL of a 10% glycerol aqueous solution, a catalyst loading of 0.5 g/L, and reaction temperature of 20 °C. Scheme S2: Reaction mechanism for glycerol photoreforming, as proposed by Sanwald et al. [39]. Reference [39] was cited in the supplementary materials.
