# Supplementary material for: Biochars from Olive Stones as Carbonaceous Support in Pt/TiO2-Carbon Photocatalysts and Application in Hydrogen Production from Aqueous Glycerol Photoreforming
Source: Nanomaterials (Basel). 2023 Apr 28;13(9):1511. doi: 10.3390/nano13091511 (PMC10179866; doi:10.3390/nano13091511)
Supplement: Supplementary file 1 [file nanomaterials-13-01511-s001.zip › nanomaterials-2341369-supplementary.pdf]

Supplementary Material

Biochars from Olive Stones as Carbonaceous Support in Pt/TiO_2-Carbon Photocatalysts and Application in Hydrogen Production from Aqueous Glycerol Photoreforming

Supplementary Scheme S1. Schematic illustration on the synthesis of the catalysts

Supplementary Figure S1. Glycerol photoreforming experiments (experimental setup)

Supplementary Figure S2. Low resolution Transmission Electron Microscopy (TEM)

Supplementary Figure S3. Scanning Transmission Electron Microscopy (STEM) – Pt/TiO_2-MCF

Supplementary Figure S4. Scanning Transmission Electron Microscopy (STEM) – Pt/TiO_2-AC

Supplementary Figure S5. Scanning Transmission Electron Microscopy (STEM) – Pt/TiO_2-PyCF

Supplementary Figure S6. UV-vis spectra

Supplementary Figure S7. Isoelectric point (IEP)

Supplementary Figure S8. Normalized particle size distribution

Supplementary Figure S9. H_2 and CO_2 evolution during photoreforming experiments

Supplementary Scheme S2. Reaction mechanism for glycerol photoreforming from Ref. [1]

Supplementary Scheme S1. Schematic illustration on the synthesis of the catalysts

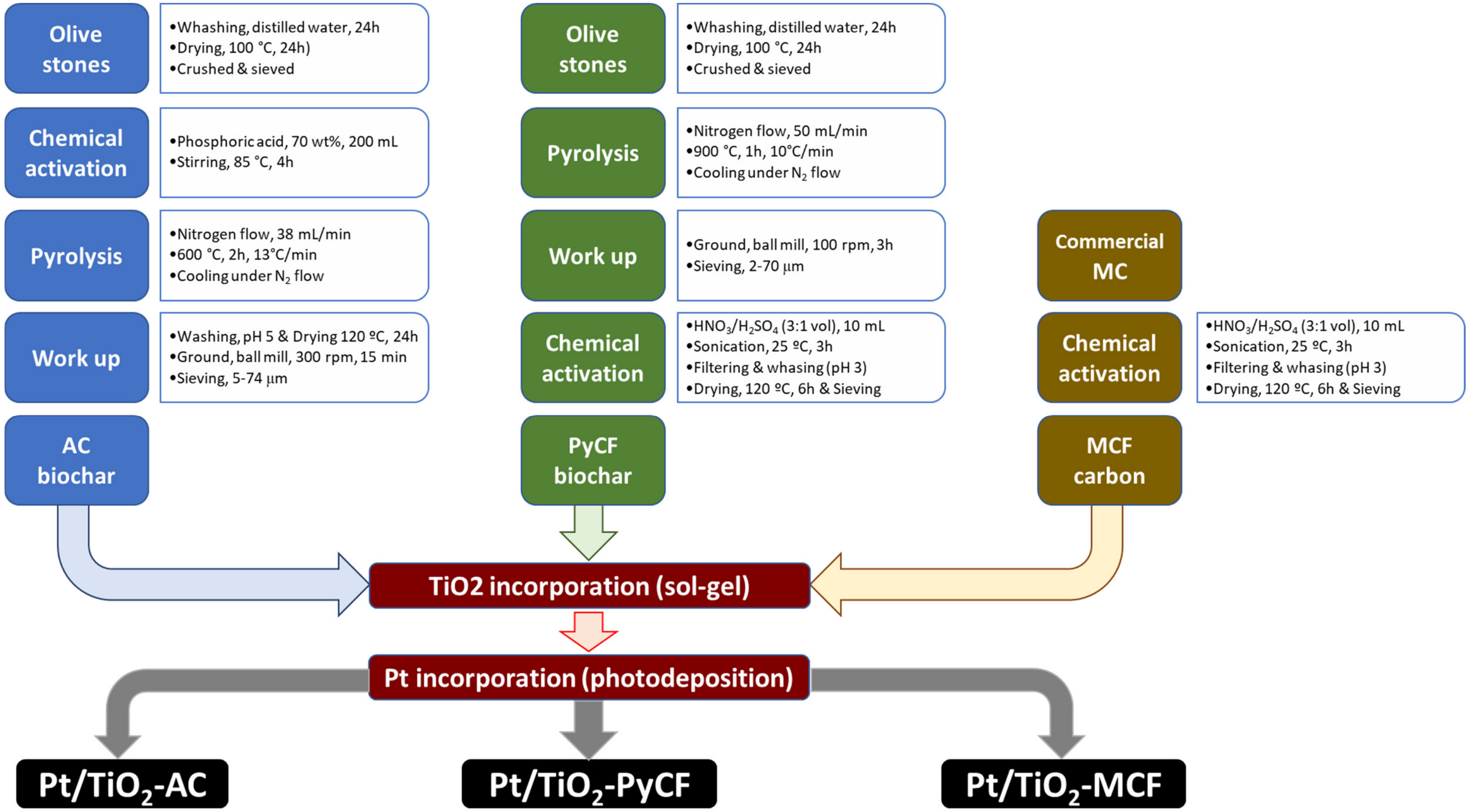

Scheme S1. Schematic illustration on the synthesis of the catalysts

Supplementary Figure S1. Glycerol photoreforming experiments (experimental setup)

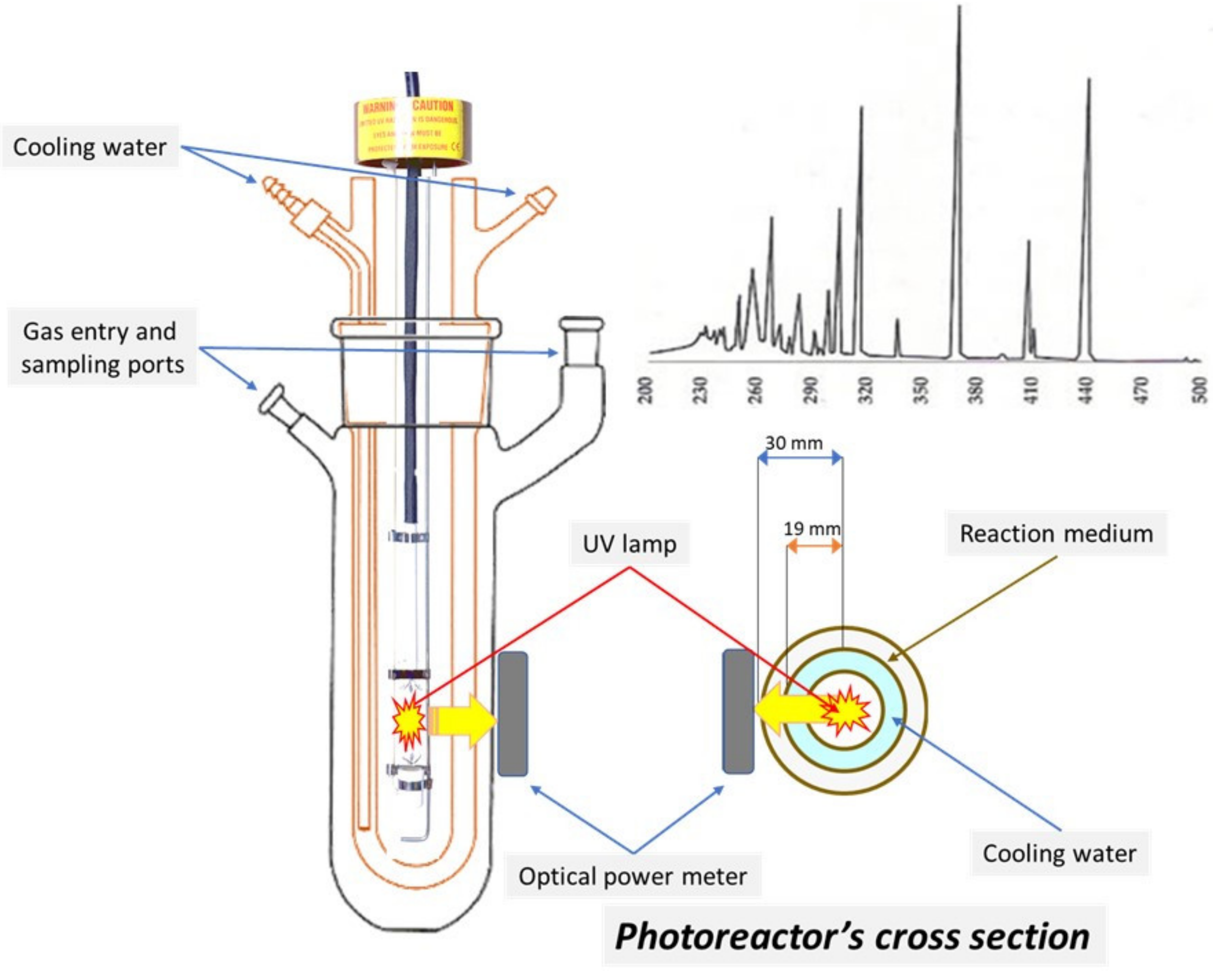

Figure S1. Experimental setup for the aqueous glycerol photoreforming experiments.

Details on UV lamp

125 Watts medium pressure mercury lamp that produces predominantly 365-366 nm radiation with smaller amounts in both the ultraviolet (at 254, 265, 270, 289, 297, 302, 313 and 334 nm) and the visible (at 405-408, 436, 546 and 577-579 nm) regions.

Details on Optical Power Meter

The radiation power emitted by the lamp was measured outside the reactor with a Newport 841-P-USAB optical power meter, equipped with a Newport 818P Series high power detector (MKS Instruments, Andover, USA).

Supplementary Figure S2. Low resolution Transmission Electron Microscopy (TEM)

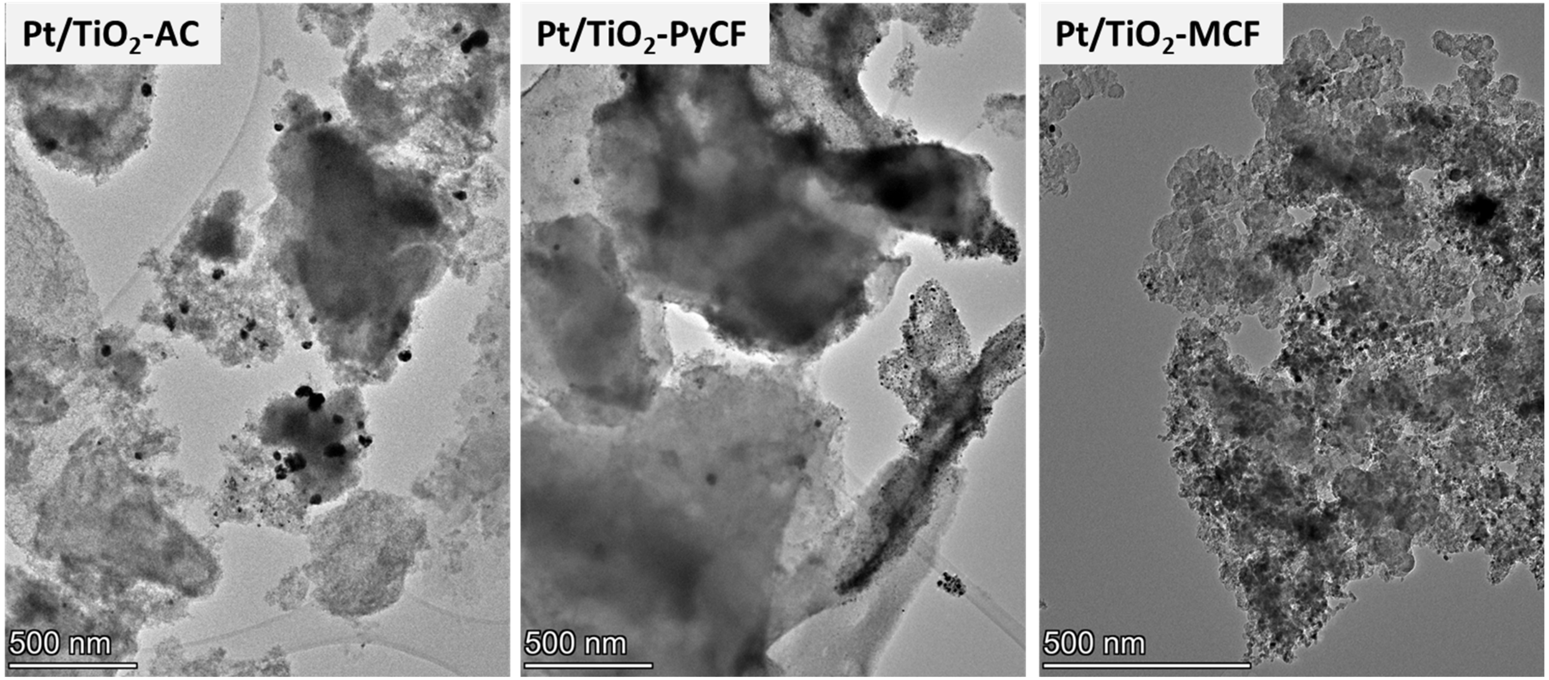

Figure S2. Morphological aspect of the synthesized photocatalysts from low resolution transmission electron microscopy images. Pt/TiO_2-AC (left), Pt/TiO_2-PyCF (center) and Pt/TiO_2-MCF (right).

Supplementary Figure S3. Scanning Transmission Electron Microscopy (STEM) – Pt/TiO_2-MCF

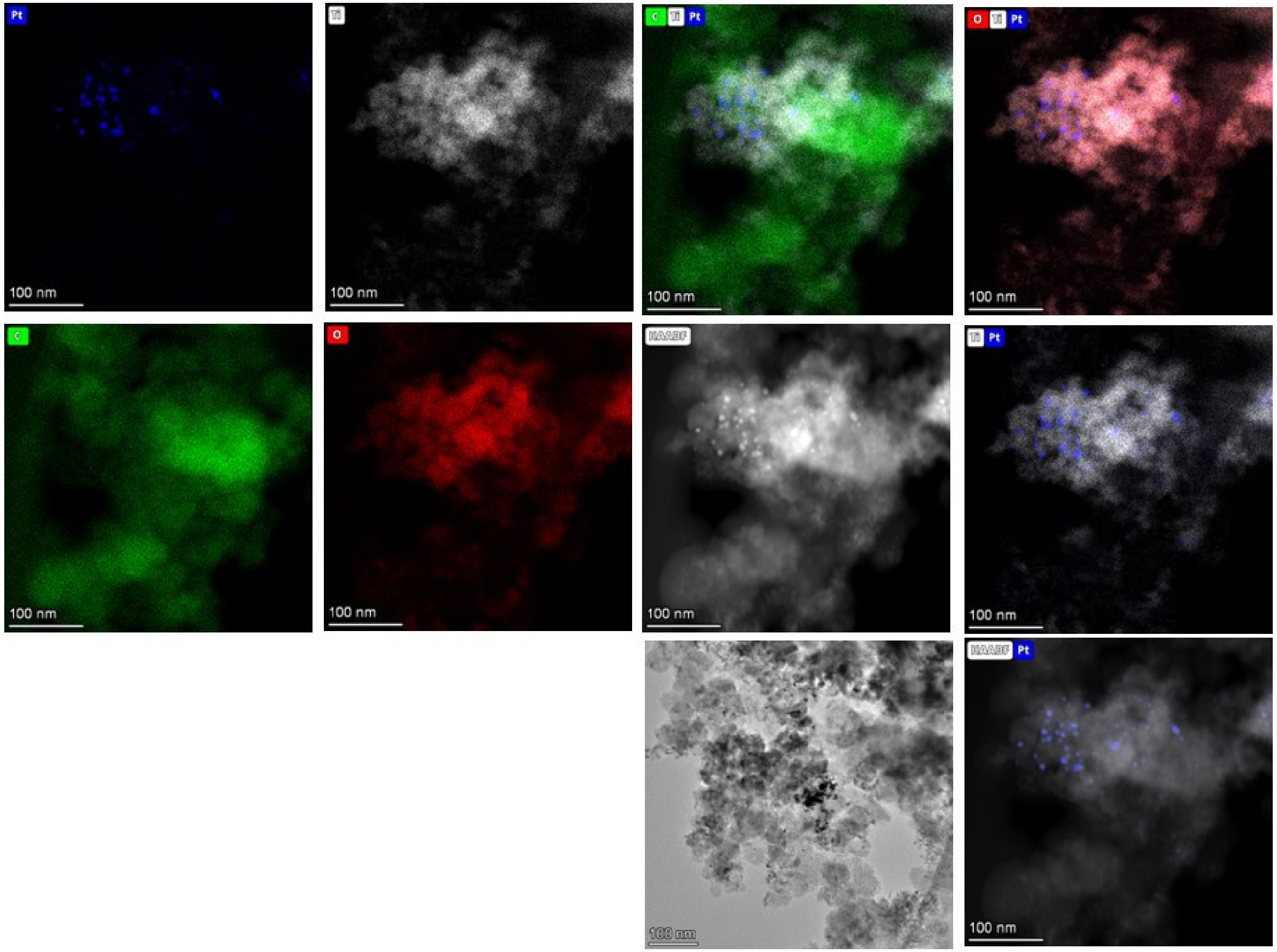

Figure S3. TEM/STEM and EDS images obtained for the Pt/TiO_2-MCF solid.

Supplementary Figure S4. Scanning Transmission Electron Microscopy (STEM) – Pt/TiO_2-AC

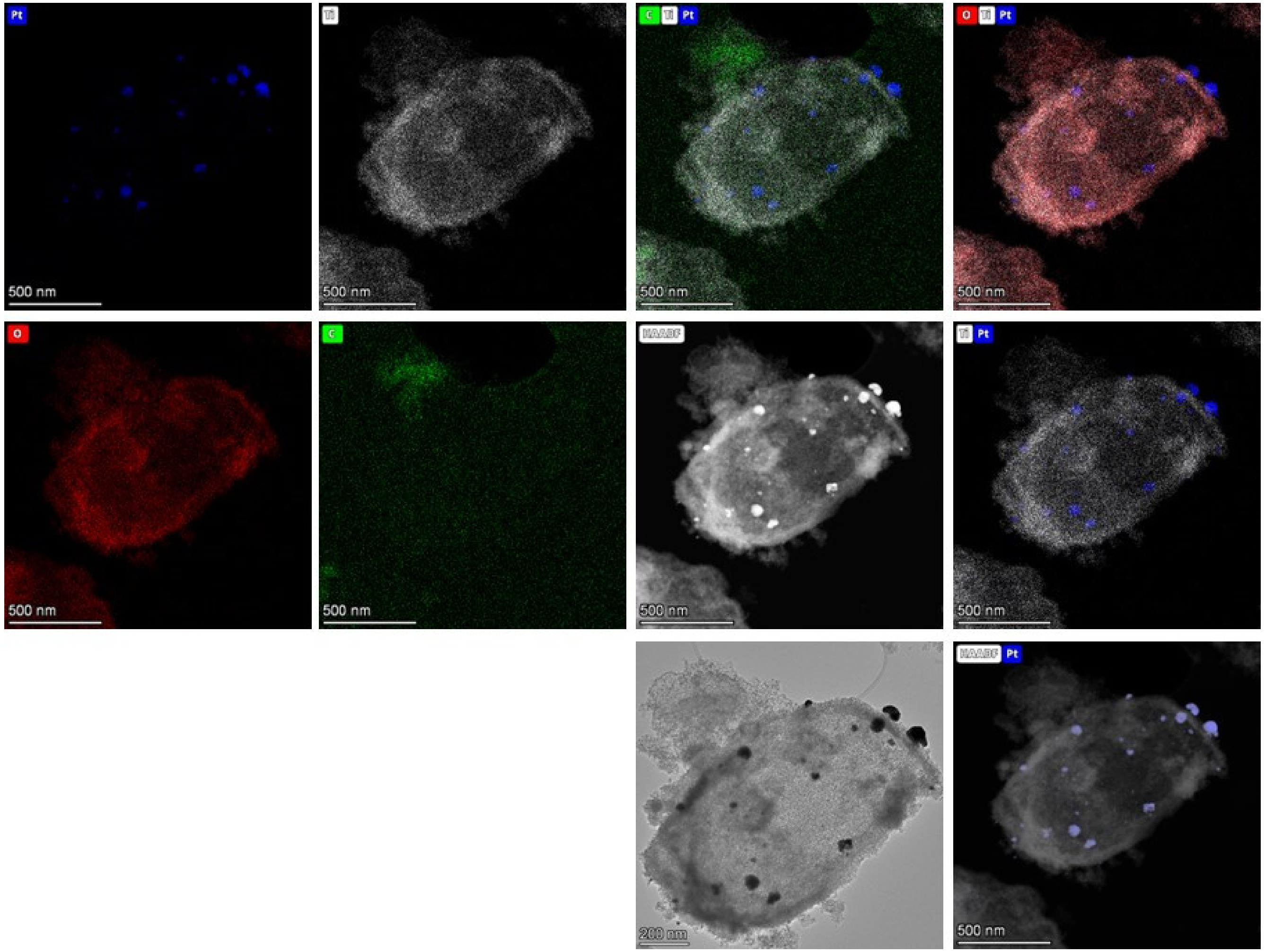

Figure S4. TEM/STEM and EDS images obtained for the Pt/TiO_2-AC solid.

Supplementary Figure S5. Scanning Transmission Electron Microscopy (STEM) – Pt/TiO₂-PyCF

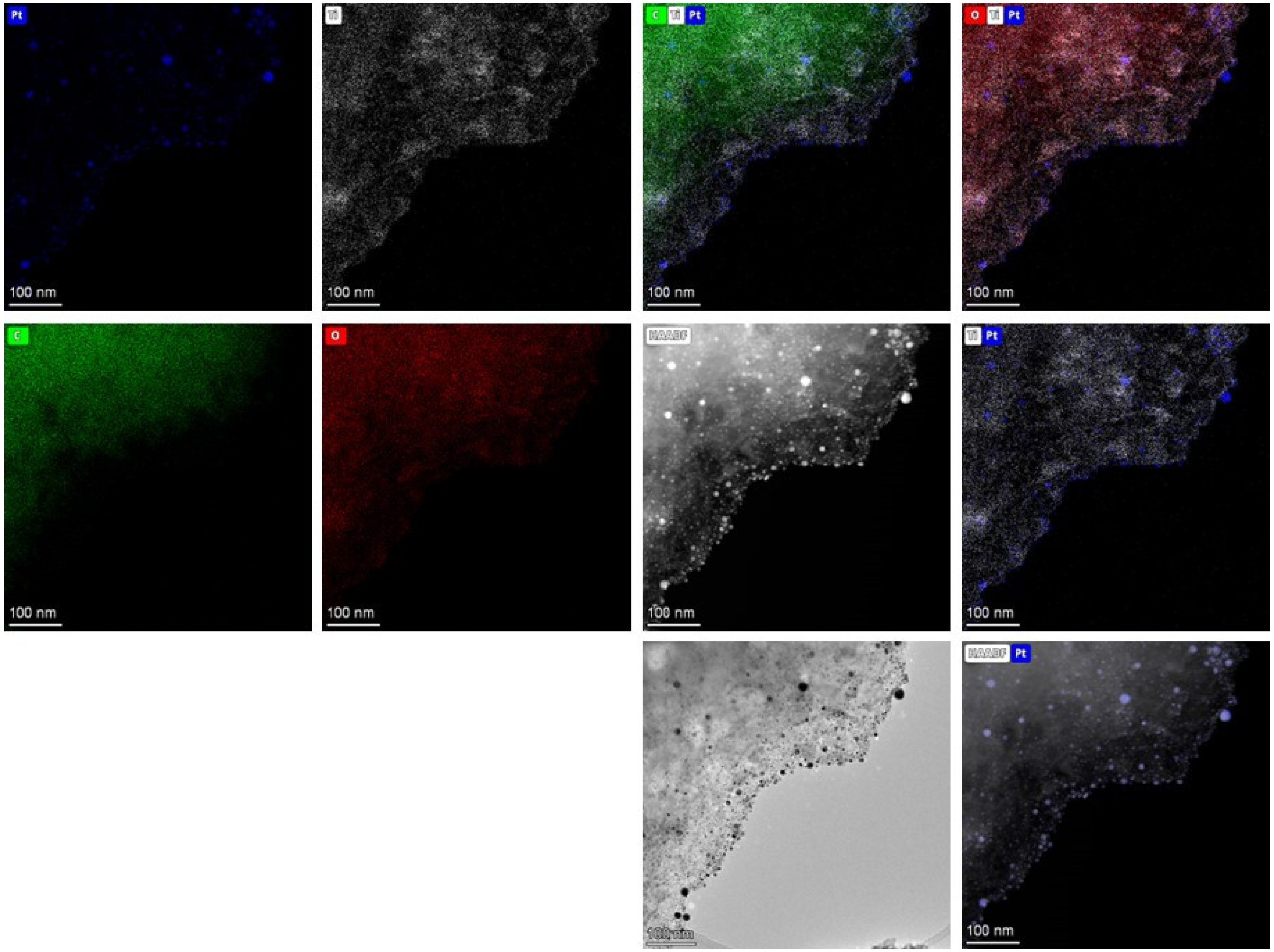

Figure S5. TEM/STEM and EDS images obtained for the Pt/TiO_2-PyCF solid.

Supplementary Figure S6. UV-vis spectra

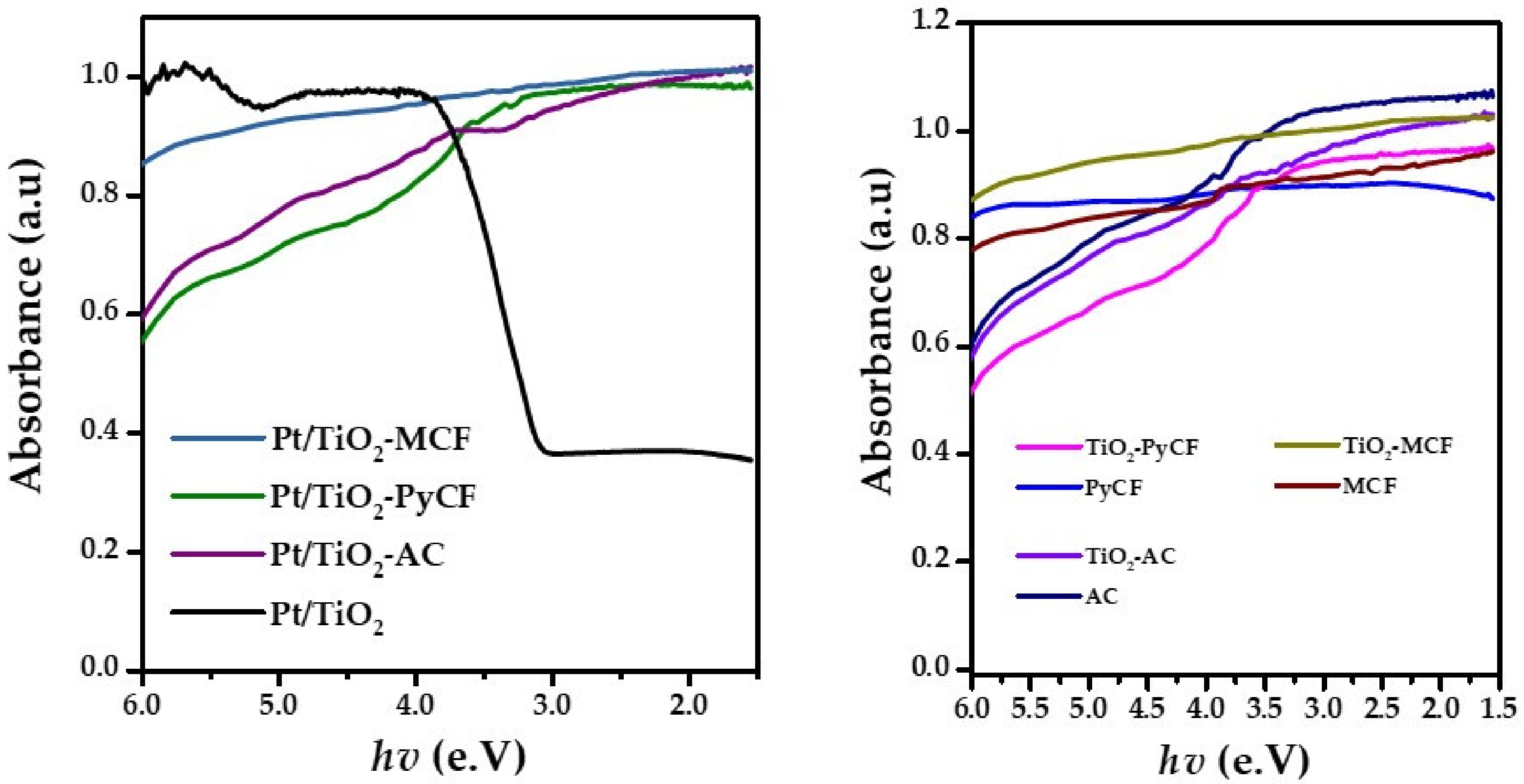

Figure S6. UV-vis spectra obtained for the Pt/TiO_2-carbon photocatalysts prepared in this work.

Supplementary Figure S7. Isoelectric point (IEP)

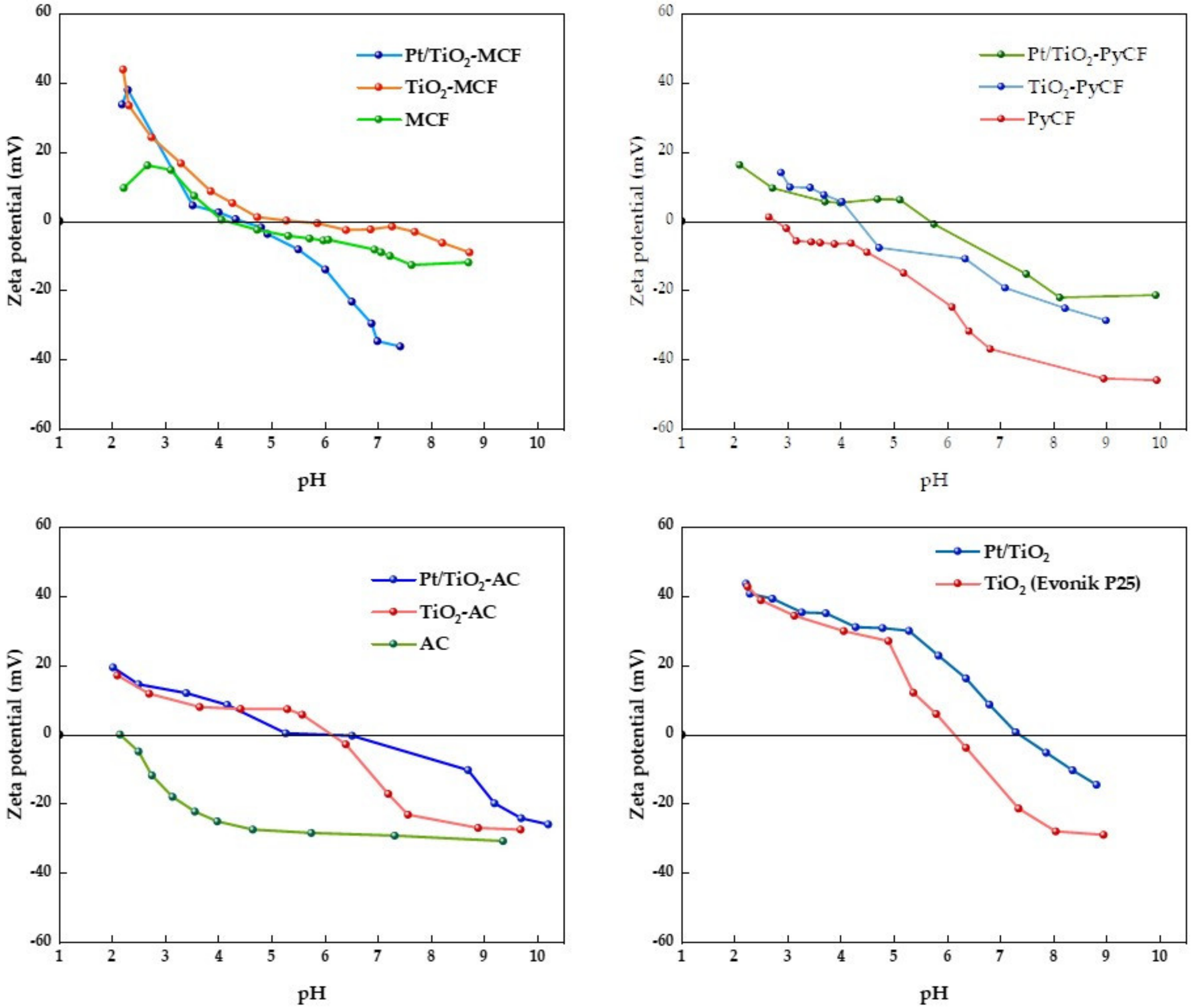

Figure S7. Isoelectric point (IEP) plot for the carbon materials, TiO_2-Carbon composites and the Pt/TiO_2-Carbon photocatalysts prepared in this work. TiO_2 Evonik P25 and Pt/TiO_2 were also included for comparison.

Supplementary Figure S8. Normalized particle size distribution

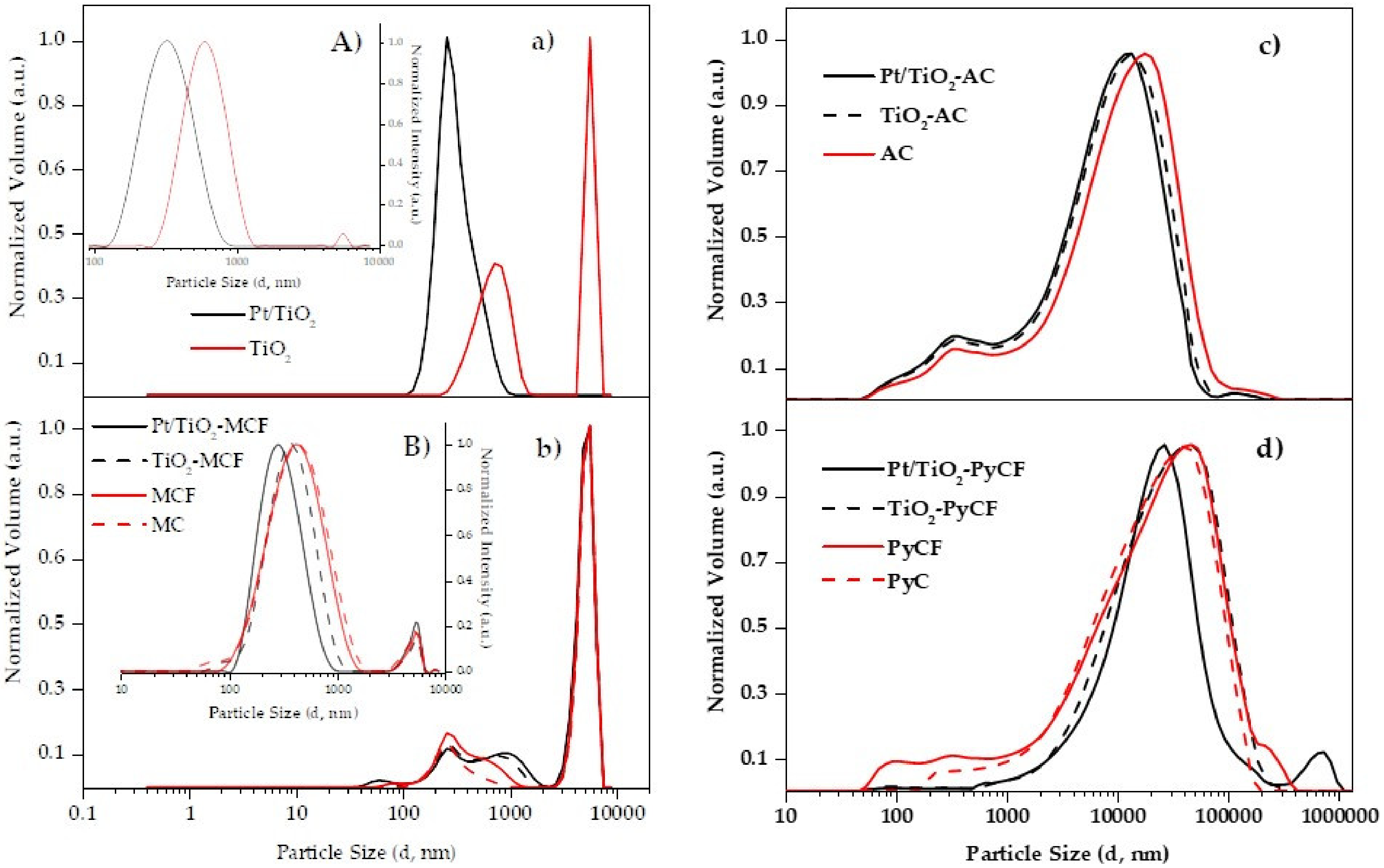

Figure S8. Normalized particle size distribution (intensity and volume) of the photocatalysts. DLS studies based on Intensity-Volume for Pt/TiO_2 (a,A) and Pt/TiO_2-MCF (b,B). LDA studies based on Volume for Pt-TiO_2-AC (c) and Pt/TiO_2-PyCF (d).

Supplementary Figure S9. H_2 and CO_2 evolution during photoreforming experiments

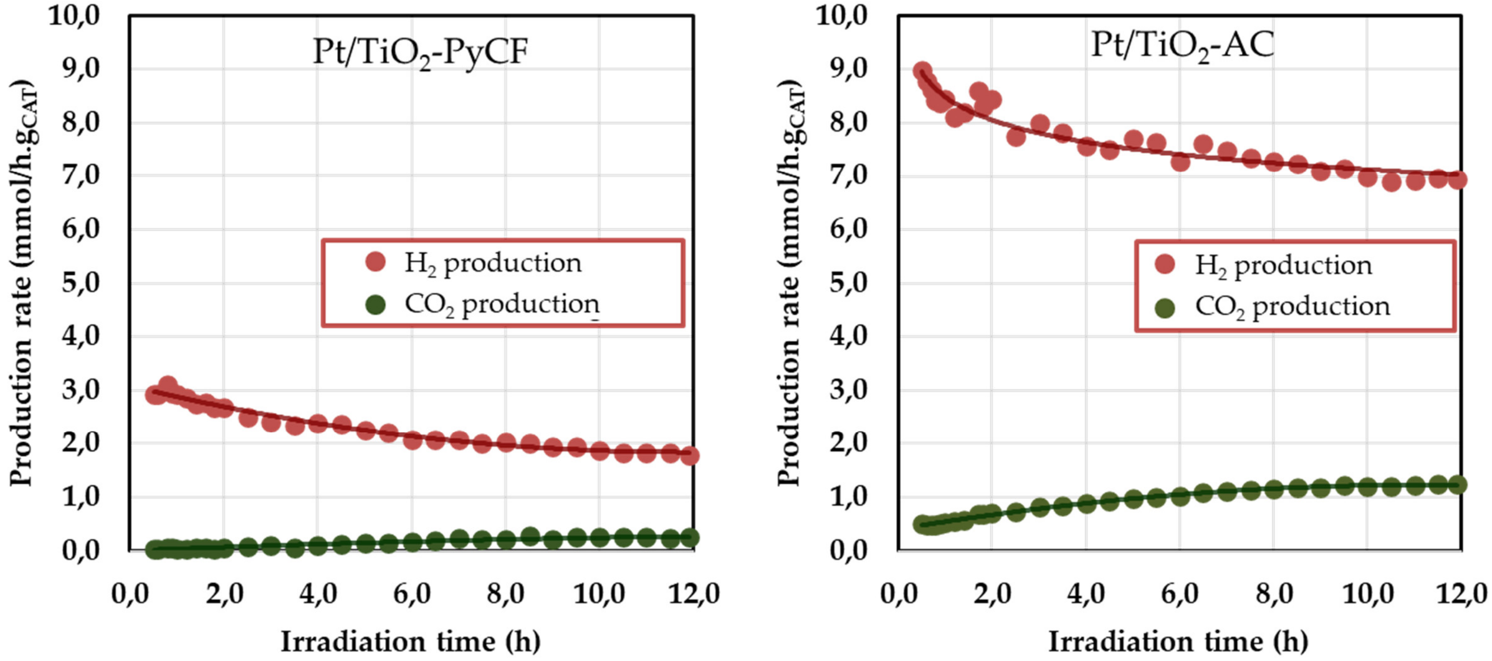

Figure S9. Hydrogen and carbon dioxide evolution during the glycerol photoreforming over Pt/TiO_2-PyCF and Pt/TiO_2-AC photocatalysts. Reactions were carried out under Ar flow (20 mL/min) with 65 mL of a 10% glycerol aqueous solution, a catalyst loading of 0.5 g/L and reaction temperature of 20°C.

Supplementary Scheme S2. Reaction mechanism for glycerol photoreforming from Ref. [1]

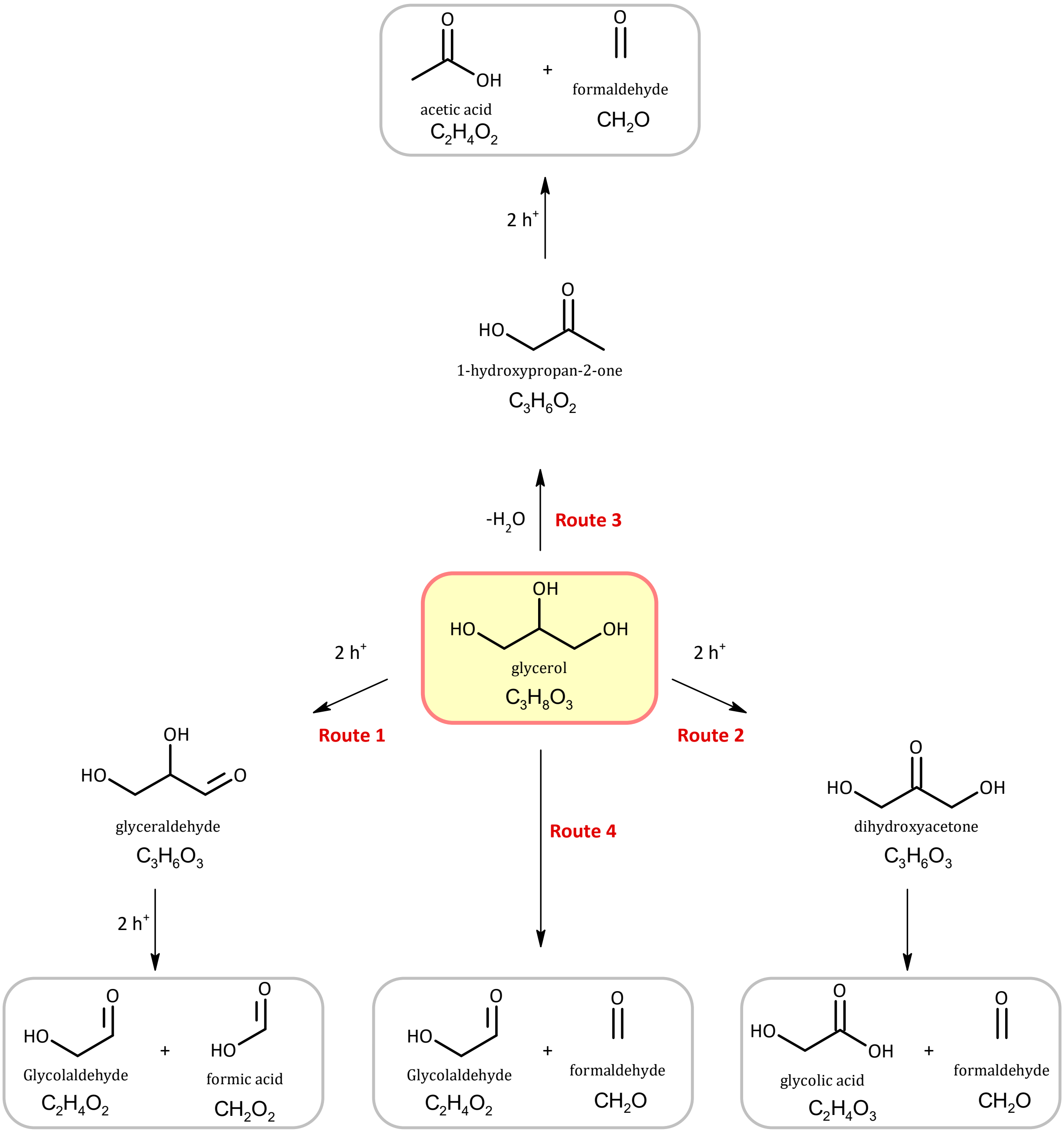

Scheme S2. Reaction mechanism for glycerol photoreforming as proposed by Sanwald et al. Ref. [1]

References

1. Sanwald, K.E.; Berto, T.F.; Eisenreich, W.; Gutiérrez, O.Y.; Lercher, J.A. Catalytic Routes and Oxidation Mechanisms in Photoreforming of Polyols. *J. Catal.* **2016**, *344*, 806–816. https://doi.org/10.1016/j.jcat.2016.08.009.
